# The lysolipid transporter Mfsd2a regulates lipogenesis in the developing brain

**DOI:** 10.1371/journal.pbio.2006443

**Published:** 2018-08-03

**Authors:** Jia Pei Chan, Bernice H. Wong, Cheen Fei Chin, Dwight L. A. Galam, Juat Chin Foo, Loo Chin Wong, Sujoy Ghosh, Markus R. Wenk, Amaury Cazenave-Gassiot, David L. Silver

**Affiliations:** 1 Signature Research Program in Cardiovascular and Metabolic Disorders, Duke-NUS Medical School, Singapore, Singapore; 2 Department of Biochemistry, National University of Singapore, Singapore, Singapore; 3 Centre for Computational Biology, Duke-NUS Medical School, Singapore, Singapore; UCSD, United States of America

## Abstract

Brain development requires a massive increase in brain lipogenesis and accretion of the essential omega-3 fatty acid docosahexaenoic acid (DHA). Brain acquisition of DHA is primarily mediated by the transporter Major Facilitator Superfamily Domain containing 2a (Mfsd2a) expressed in the endothelium of the blood-brain barrier (BBB) and other abundant cell types within the brain. Mfsd2a transports DHA and other polyunsaturated fatty acids (PUFAs) esterified to lysophosphatidylcholine (LPC-DHA). However, the function of Mfsd2a and DHA in brain development is incompletely understood. Here, we demonstrate, using vascular endothelial-specific and inducible vascular endothelial-specific deletion of *Mfsd2a* in mice, that Mfsd2a is uniquely required postnatally at the BBB for normal brain growth and DHA accretion, with DHA deficiency preceding the onset of microcephaly. In Mfsd2a-deficient mouse models, a lipidomic signature was identified that is indicative of increased de novo lipogenesis of PUFAs. Gene expression profiling analysis of these DHA-deficient brains indicated that sterol regulatory-element binding protein (Srebp)-1 and Srebp-2 pathways were highly elevated. Mechanistically, LPC-DHA treatment of primary neural stem cells down-regulated Srebp processing and activation in a Mfsd2a-dependent fashion, resulting in profound effects on phospholipid membrane saturation. In addition, Srebp regulated the expression of Mfsd2a. These data identify LPC-DHA transported by Mfsd2a as a physiological regulator of membrane phospholipid saturation acting in a feedback loop on Srebp activity during brain development.

## Author summary

The brain is the most lipid-rich organ in the body. Brain development involves a tremendous increase in the synthesis and accretion of fatty acids. De novo synthesis of fatty acids is mediated by Srebp transcription factors, whereas acquisition of essential fatty acids via uptake of plasma-derived lysophosphatidylcholine containing the essential omega-3 fatty acid docosahexaenoic acid (LPC-DHA) is mediated by the transporter Mfsd2a in the cells that line the blood vessels in the brain. The function of Mfsd2a and DHA in brain development is incompletely understood. Our study determined that Mfsd2a is required at the blood-brain barrier for brain development and accretion of DHA after birth in mice. Moreover, we determined that a major function of DHA in the brain is to negatively regulate Srebp activation, resulting in profound effects on membrane phospholipid composition. These findings reveal that LPC-DHA transported by Mfsd2a plays a physiological role in both brain growth and in maintaining plasma membrane phospholipid composition during brain development.

## Introduction

The brain is one of the most lipid-rich organs in the body, consisting mostly of glycerophospholipids, cholesterol, and sphingolipids [1]. Prenatal brain development is a complex developmental process that involves the coordinated establishment of hundreds of specialized cell types and the building of synaptic connectivity, together with a functioning blood-brain barrier (BBB) [2–4]. Postnatal brain growth involves proliferation of astrocytes and oligodendrocytes, myelination of axons, and expansion of neuron dendrites. A common and essential requirement for both prenatal and postnatal brain development is the biosynthesis of a massive amount of membrane phospholipid, the origin of which was believed to be exclusively derived from de novo biosynthesis within cells of the brain, and acquisition of essential fatty acids from the periphery into the developing brain. De novo lipogenesis is driven by sterol regulatory-element binding proteins (SREBPs), identified by Brown and Goldstein to be transcription factors that are important for the regulation of genes that maintain cellular lipid homeostasis. There are 3 known isoforms—Srebp-1a, -1c, and Srebp-2; Srebp-1c regulates genes in fatty acid biosynthesis, Srebp-2 regulates genes involved in cholesterol biosynthesis, and Srebp-1a regulates both [5]. Srebp-1c and -2 predominate in the brain [6]. In the presence of cholesterol or oxysterols, the precursor form of Srebp (about 130 kDa), in complex with its chaperone protein sterol cleavage-activating protein (Scap), is retained in the endoplasmic reticulum (ER) bound to insulin-induced gene 1 (Insig-1) [7]. In the absence of sterols, the Srebp-Scap complex is released from Insig-1 and transported from the ER to the Golgi apparatus on COPII vesicles [8]. At the Golgi, the Srebp precursor is cleaved by Site-1 and Site-2 proteases, releasing the N-terminal basic helix-loop-helix leucine zipper transcription factor domain (about 60–70 kDa) that enters the nucleus and activates target genes by binding to sterol regulatory elements (SREs) [9]. Underscoring the importance of de novo lipogenesis for brain development, targeted deletion of Scap in brain resulted in perinatal lethality [10].

Docosahexaenoic acid (DHA) is an omega-3 fatty acid that is highly enriched in brain phospholipids, particularly in phosphatidylethanolamine (PE), phosphatidylserine (PS), and phosphatidylcholine (PC) pools and comprises up to 15% or more of the total fatty acid composition of the prefrontal cortex [11,12]. During human fetal development, a rapid increase in brain DHA level is reported to occur at the end of the second trimester, which coincides with the development of the BBB [13,14]. The accretion of DHA in brain continues from early postnatal days until approximately 2 years of age [15–17]. Decreased levels of DHA in the developing brain have been linked with negative effects on cognitive function [18,19] and neurodevelopmental disorders [20–22]. Importantly, DHA itself cannot be de novo synthesized efficiently in brain and must be transported across the BBB. Our laboratory discovered Major Facilitator Superfamily Domain containing 2a (Mfsd2a), a sodium-dependent lysophosphatidylcholine (LPC) transporter highly expressed by the endothelium of the BBB and the blood-retinal barrier (BRB), to be the major pathway for brain and eye DHA accretion [23,24]. Mfsd2a does not transport unesterified DHA, but DHA esterified as lysophosphatidylcholine (LPC-DHA). Mice with gene-targeted deletion of *Mfsd2a* (2aKO) exhibited severe microcephaly and brain DHA deficiency [23,25]. Importantly, we have also identified 3 families with homozygous nonsynonymous loss-of-function mutations in *Mfsd2a* that presented with severe microcephaly and intellectual disability [26,27]. Moreover, plasma LPC levels were increased in affected patients relative to controls and, similar to 2aKO mice, consistent with a lack of brain uptake of LPCs. Collectively, these findings suggested that brain growth and function was impaired in patients because of the lack of transport of LPCs, and likely in part because of brain deficiency in DHA. Despite the abundance and importance of DHA in the developing brain, very little is known about its biochemical and molecular function. In the current study, we provide genetic and biochemical evidence that Mfsd2a is required at the BBB during postnatal life to mediate normal brain growth and that DHA deficiency precedes the onset of microcephaly. Moreover, using an unbiased gene-profiling approach, we determined that a major function of DHA during brain development is to regulate Srebp-1 and Srebp-2 activity resulting in major changes in phospholipid saturation.

## Results

### Mfsd2a is required at the BBB for postnatal brain growth

**Fig 1:**
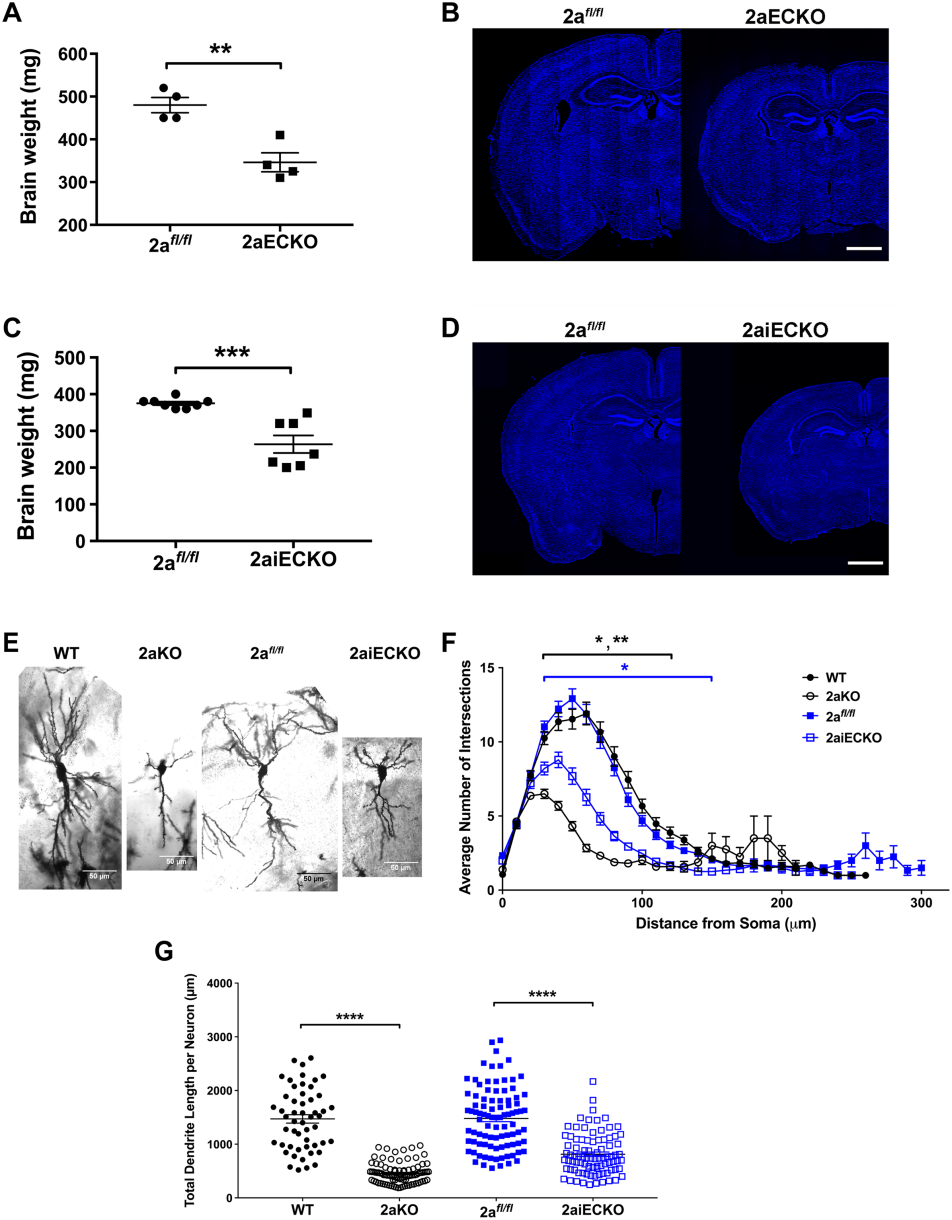
Mfsd2a is required at the BBB for postnatal brain growth. (A) Reduced brain weights of 8-week-old 2aECKO relative to 2a^*fl/fl*^ mice. Data are represented as mean ± SE. 2a^*fl/fl*^, *n* = 4; 2aECKO, *n* = 4. **p < 0.01. (B) Coronal sections indicate reduced brain size of 8-week-old 2aECKO relative to 2a^*fl/fl*^ mice. Sections were stained with Hoechst. Scale bar, 1 mm. (C) Reduced brain weights of 4-week-old 2aiECKO relative to 2a^*fl/fl*^ mice. Data are represented as mean ± SE. 2a^*fl/fl*^, *n* = 8; 2aiECKO, *n* = 7. ***p < 0.001. (D) Coronal sections indicate reduced brain size of 4-week-old 2aiECKO relative to 2a^*fl/fl*^ mice. Sections were stained with Hoechst. Scale bar, 1 mm. (E) Representative images of Golgi-stained hippocampal neurons in brain sections of 3-week-old WT and 2aKO, and 4-week-old 2a^*fl/fl*^ and 2aiECKO mice. *n* = 2 of each genotype. Scale bar = 50 μm. (F) Sholl analysis of Golgi-stained hippocampal neurons in sections of brains from 3-week-old WT and 2aKO, and 4-week-old 2a^*fl/fl*^ and 2aiECKO mice. An average of 44 neurons were analyzed for each brain. Data are represented as mean ± SE. *n* = 2 of each genotype. 2aKO neurons were significantly shorter relative to WT between 30 and 120 μm from the soma. 2aiECKO neurons were significantly shorter relative to 2a^*fl/fl*^ between 30 and150 μm from the soma. *p < 0.05; **p < 0.01. (G) Total dendrite length per Golgi-stained hippocampal neuron in sections of brains from 3-week-old WT and 2aKO, and 4-week-old 2a^*fl/fl*^ and 2aiECKO mice. An average of 82 neurons were analyzed for each brain. Data are represented as mean ± SE. *n* = 2 of each genotype. ****p < 0.001. Numerical values underlying panels 1A, C, F, and G can be found in S1 Data.

We previously found that the brain sizes of conventional gene-targeted Mfsd2a knockout mice (2aKO) at embryonic day 18.5 (e18.5), a stage equivalent to late third trimester in human development, were similar to wild-type (WT) littermates, and the brains were DHA deficient [23]. These findings suggest that DHA deficiency precedes the development of microcephaly in 2aKO brain and that the development of microcephaly occurs postnatally. While Mfsd2a expression is highest in endothelial cells of the BBB, Mfsd2a is expressed in other cell types within the brain parenchyma—notably the oligodendrocyte lineage, and at lower levels in astrocytes (S1A Fig, [28]; S1B Fig, [29]). To determine if Mfsd2a deficiency at the BBB endothelium is responsible for microcephaly reported in 2aKO mice, an endothelial-specific deletion of Mfsd2a was generated using a floxed allele of Mfsd2a (2a^*fl/fl*^) crossed to the endothelial cre-driver line Tie2 (2aECKO). Brain weights of adult 2aECKO mice were significantly reduced relative to control floxed mice (2a^*fl/fl*^, Fig 1A), consistent with reduced brain size (Fig 1B). This reduction in brain size in 2aECKO mice was similar to the microcephaly of 2aKO mice [23] and indicates that Mfsd2a in the BBB is essential for brain development.

To determine if Mfsd2a at the BBB is required for postnatal brain development, we generated a tamoxifen-inducible endothelial-specific Mfsd2a deletion mouse model using the Cdh5ERT2-cre driver line crossed to 2a^*fl/fl*^ mice (2aiECKO). Deletion of Mfsd2a was induced by tamoxifen injection of pups for 3 consecutive days beginning at the day of birth, and brains were harvested at 4 weeks of age (injection scheme, S2A Fig), a time when brain growth is largely completed. Immunofluorescence imaging indicated reduced Mfsd2a at the BBB in 2aiECKO mice relative to controls (S2B Fig). Brain weights and sizes of 2aiECKO mice were significantly reduced relative to controls (Fig 1C and 1D). Because neurogenesis and cortical patterning are completed before birth, prenatal causes of microcephaly commonly result in changes in cortical patterning, while postnatal causes of microcephaly often result in decreased neuron arborization (i.e., decreased neuron dendrite branching) [30]. Remarkably, despite the reduction in size of the adult brain cortex of 2aKO mice, cortical patterning of 2aKO is similar to WT brains (S3 Fig). In addition, the cortex of brains of 2aKO mice at e18.5, prior to the development of microcephaly, also showed normal cortical patterning (S4 Fig). Consistent with a role of Mfsd2a in postnatal brain growth, brains of 2aKO and 2aiECKO exhibited significantly reduced neuronal arborization and decreased dendrite length (Fig 1E, 1F and 1G). These data indicate that Mfsd2a is required for postnatal brain growth and are consistent with the conclusion that LPC transport into the brain provides lipid for the rapidly expanding brain.

### Mfsd2a expression at the BBB is indispensable for DHA accretion during postnatal brain growth

**Fig 2:**
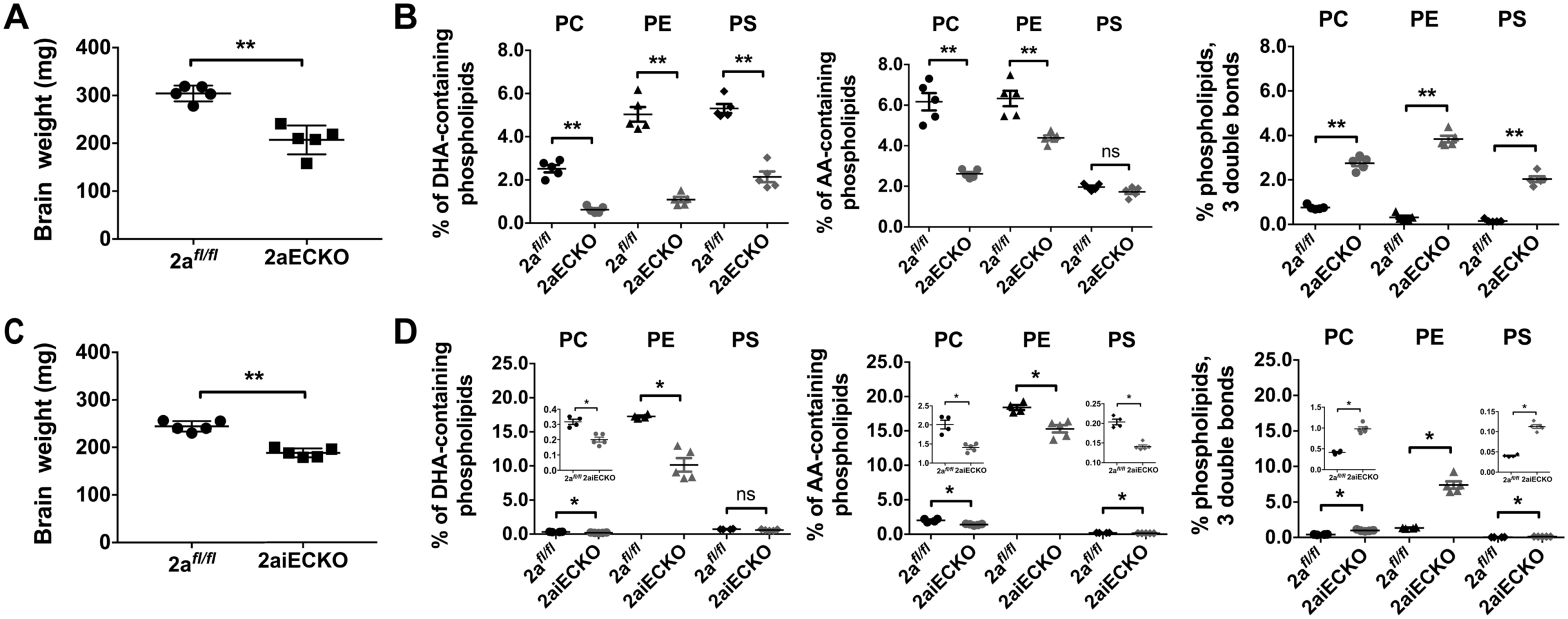
Mfsd2a expression at the BBB is indispensable for DHA accretion during postnatal brain growth. (A) Reduced brain weights of P8 2aECKO relative to 2a^*fl/fl*^ mice. Data are represented as mean ± SE. 2a^*fl/fl*^, *n* = 5; 2aECKO, *n* = 5. **p < 0.01. (B) Targeted lipidomic analysis of brains from P8 2a^*fl/fl*^ and 2aECKO mice. Percentage PC, PE, and PS containing DHA, AA, and phospholipids with both fatty acids having a cumulative 3 double bonds are shown. Data are represented as mean ± SE. 2a^*fl/fl*^, *n* = 5; 2aECKO, *n* = 5; biological replicates. **p < 0.01. (C) Reduced brain weights of P8 2aiECKO relative to 2a^*fl/fl*^ mice. Data are represented as mean ± SE. 2a^*fl/fl*^, *n* = 5; 2aiECKO, *n* = 5. **p < 0.01. (D) Targeted lipidomic analysis of brains from P8 2a^*fl/fl*^ and 2aiECKO mice. Percentage PC, PE, and PS containing DHA, AA, and phospholipids with both fatty acids having a cumulative 3 double bonds are shown. Data are represented as mean ± SE. Inset contains enlarged scatterplots when differences between groups are obscured by the scale of the y-axis. 2a^*fl/fl*^, *n* = 4; 2aiECKO, *n* = 5; biological replicates. *p < 0.05. Experimental data depicted in this figure can be found in S1 Data.

Given that Mfsd2a is required at the BBB for postnatal brain growth, we tested whether Mfsd2a at the BBB is also required for postnatal brain DHA accretion. We took a targeted lipidomic approach to quantify major DHA-containing brain phospholipids in PC, PE, and PS in the rapidly growing postnatal day 8 (P8) brain. Consistent with the development of postnatal microcephaly, 2aECKO and 2aiECKO brains were already significantly smaller at P8 relative to 2a^*fl/fl*^ brains (Fig 2A and 2C). Targeted lipidomic analysis of 2aECKO demonstrated a profound 70% decrease in DHA-containing phospholipids in the combined PC, PE, and PS pools (Fig 2B, heatmaps illustrating percentage of individual PC, PE, and PS phospholipid species shown in S5 Fig). We have previously shown that 2aKO mice exhibited a significant increase in arachidonic acid (AA)-containing brain phospholipids [23]. However, 2aECKO brains had a 40% decrease in AA-containing phospholipids in the combined PC, PE, and PS pools (Fig 2B and S5 Fig), suggesting that increased AA observed in brains of 2aKO was the result of Mfsd2a deficiency at the BBB combined with deficiency in the brain parenchyma. Notably, a significant 6-fold increase in the combined PC, PE, and PS pools containing a fatty acid sum composition of 3 double bonds was observed in 2aECKO brains (Fig 2B and S5 Fig). An increase in fatty acid desaturation is intriguing, because these fatty acid species are the products of fatty acid desaturases (i.e., SCD and FADS) that are rate-limiting enzymes in mono- and polyunsaturated fatty acid (PUFA) biosynthesis. Increases in these particular fatty acid species in 2aECKO brains were also noted in 2aKO mice [23]. Performing the same targeted lipidomic analysis on P8 brains of 2aiECKO and controls revealed that 2aiECKO brains had a significant 40% reduction in DHA-containing phospholipids in the combined PC and PE pools and an 18% reduction in AA-containing phospholipids in the combined PC, PE, and PS pools, albeit at a lower magnitude difference relative to 2aECKO brains. Similar to 2aECKO brains, a significant 4-fold increase in the combined PC, PE, and PS pools containing a fatty acid sum composition of 3 double bonds was observed in 2aiECKO brains (Fig 2D, heatmaps illustrating percentage of individual PC, PE, and PS phospholipid species shown in S6 Fig). These lipidomic analyses indicated that Mfsd2a plays an essential role for DHA accretion at the BBB during the rapid phase of postnatal brain growth. In addition, Mfsd2a deficiency at the BBB resulted in a lipidomic signature suggestive of increased de novo lipogenesis of mono- and polyunsaturated fatty acids.

### De novo lipogenesis pathways are up-regulated in DHA-deficient brains

**Fig 3:**
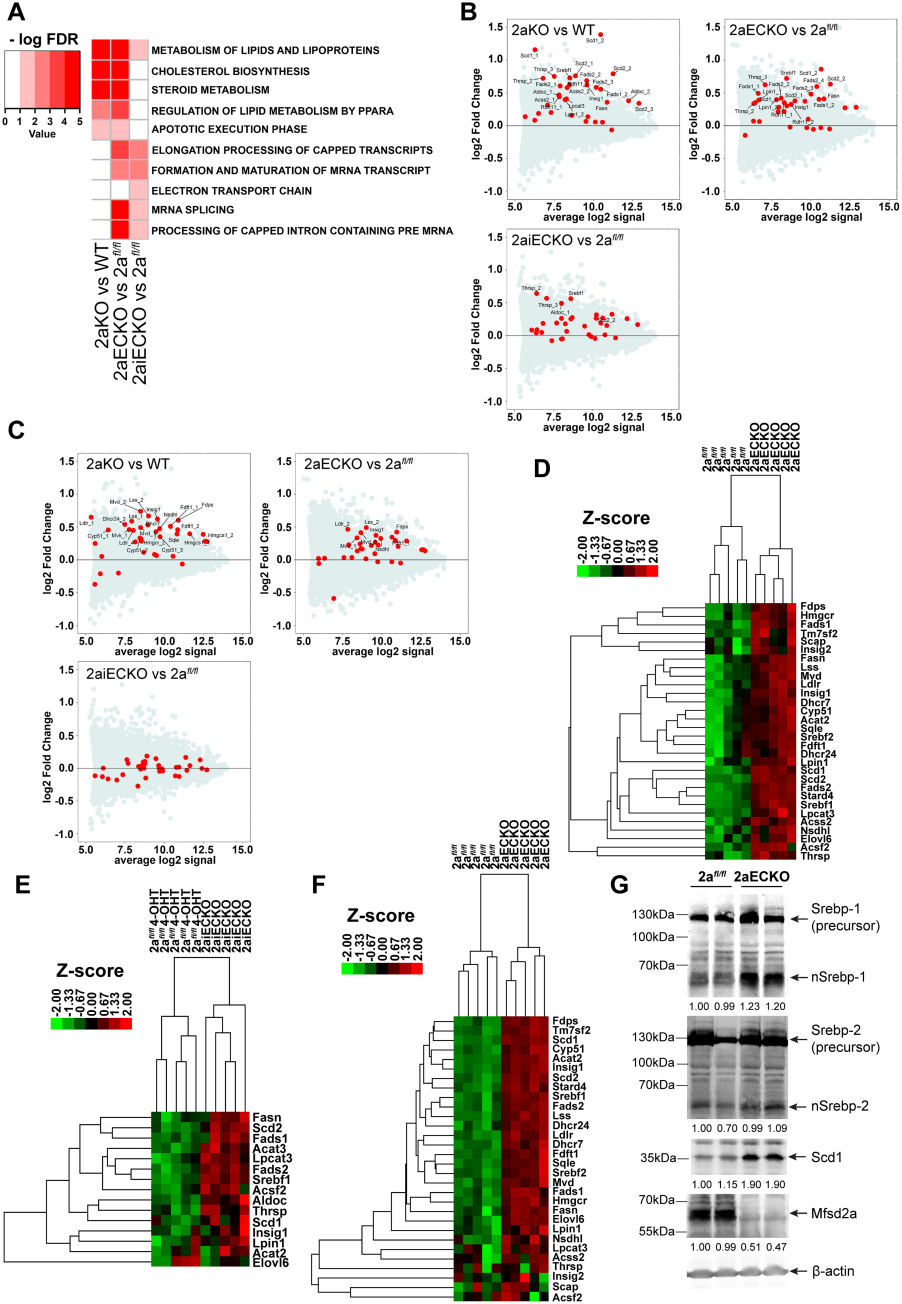
De novo lipogenesis pathways are up-regulated in DHA-deficient brains. (A) Gene microarray analysis of brains from P8 mice. Heatmap represents commonly up-regulated Reactome pathways with an FDR ≤5%. Only pathways that are commonly up-regulated in at least 2 genotype comparisons (2aKO versus WT or 2a^*fl/fl*^ versus 2aECKO/2aiECKO) are shown. RNA was pooled from 5–6 cerebrums according to their genotype. Color key indicates negative logarithm of the FDR for the selected pathways, for which a higher value indicates the pathway is more significantly different. (B) Increased Srebp-1 pathway genes in Mfsd2a deficiency mouse models. Data are represented as MA plots of gene microarray analysis of brains from P8 mice. MA plot is used to visualize intensity-dependent ratio of raw gene microarray data and indicated that the top-ranked pathway that was up-regulated in 2aKO, 2aECKO, and 2aiECKO was the metabolism of lipids and lipoproteins pathway, which contains lipogenic targets of Srebf1 (Srebp-1). Red dots represent individual targets with significant changes in this pathway over the background of expressed genes. (C) Increased Srebp-2 pathway genes in Mfsd2a deficiency mouse models. MA plot of gene microarray analysis of brains from P8 mice. The second highest ranked pathway that was up-regulated in 2aKO and 2aECKO was the cholesterol biosynthesis and steroid metabolism pathway, which contains cholesterogenic targets of Srebf2 (Srebp-2). Red dots represent individual targets with significant changes in this pathway over the background of expressed genes. (D) Confirmation of up-regulation of Srebp-1 and Srebp-2 pathways using direct mRNA quantification by Nanostring analysis on brains from P8 2a^*fl/fl*^ and 2aECKO mice. Heatmap represents agglomerative hierarchical clustering based on Euclidean distance using normalized counts of Srebp-1 and Srebp-2 gene targets. Color bar indicates z-score transformations on genes using normalized counts. 2a^*fl/fl*^, *n* = 5; 2aECKO, *n* = 5; biological replicates. (E) Confirmation of up-regulation of Srebp-1 pathway using direct mRNA quantification by Nanostring analysis on brains from P8 2a^*fl/fl*^ and 2aiECKO mice, in which deletion of Mfsd2a in the BBB was induced by daily injections of 4-OHT injections from postnatal day 0 to 3. Heatmap represents agglomerative hierarchical clustering based on Euclidean distance using normalized counts of Srebp-1 gene targets. Color bar indicates z-score transformations on genes using normalized counts. 2a^*fl/fl*^, *n* = 5; 2aiECKO, *n* = 5; biological replicates. (F) Nanostring analysis on Srebp-1 and Srebp-2 gene targets from brains from e18.5 2a^*fl/fl*^ and 2aECKO mice showed up-regulation of both pathways in 2aECKO relative to 2a^*fl/fl*^. Heatmap represents agglomerative hierarchical clustering based on Euclidean distance using normalized counts of Srebp-1 and Srebp-2 gene targets. Color bar indicates z-score transformations on genes using normalized counts. 2a^*fl/fl*^, *n* = 5; 2aECKO, *n* = 5; biological replicates. (G) Western blot analysis of Srebp-1, Srebp-2, Scd1, and Mfsd2a expression in brain lysates of P13 mice. 2aECKO brains had increased nSrebp-1 relative to 2a^*fl/fl*^ brains. nSrebp-2 levels were similar between genotypes. Scd1, a readout of Srebp-1 activity, was more abundant in brains of 2aECKO relative to 2a^*fl/fl*^ mice. Mfsd2a expression was highly reduced in 2aECKO relative to 2a^*fl/fl*^ brains. Quantification of nSrebp-1, nSrebp-2, Scd1, and Mfsd2a is shown. β-actin served as a loading control. Numerical values underlying panel 3G can be found in S1 Data.

Mfsd2a knockout mice (i.e., 2aKO, 2aECKO, 2aiECKO) are the first genetic models of brain DHA deficiency without the need to feed mice omega-3 fatty acid–deficient diets. We reasoned that Mfsd2a deficiency models could be a suitable tool to identify biochemical pathways that are regulated by DHA, thus revealing functions of DHA in the growing brain. To exploit Mfsd2a deficiency models for this purpose, transcriptome analysis using gene microarrays was performed on the cerebrums of P8 or P9 2aKO, 2aECKO, and 2aiECKO and their respective age-matched controls (WT or 2a^*fl/fl*^). Remarkably, the top predicted Reactome pathway for up-regulated genes in brains of 2aKO, 2aECKO, and 2aiECKO was the metabolism of lipids and lipoproteins pathway that contains lipogenic targets of the transcription factor Srebp-1 (Fig 3A). In addition, the cholesterol biosynthesis and steroid metabolism pathways were up-regulated in brains of 2aKO and 2aECKO, which contain targets of the transcription factor Srebp-2 (Fig 3A). MA plots were used to visualize the mean expression versus fold-change relationship for all genes, with individual targets in the Srebp-1 and Srebp-2 pathways indicated as red circles (Fig 3B and 3C, respectively). It is also interesting to note that these findings are consistent with the top 2 predicted up-regulated pathways in eyes from 2aKO mice, which are also DHA deficient [24], suggesting that this result is likely a common adaptive response to Mfsd2a deficiency. Validation of increased Srebp-1 and Srebp-2 target gene expression was carried out by direct quantification of mRNA using Nanostring analysis. Consistent with the gene microarray findings, brains of 2aECKO mice had up-regulation of Srebp-1 and Srebp-2 target genes relative to controls, while only Srebp-1 target genes were up-regulated in brains of 2aiECKO mice (Fig 3D and 3E, respectively; see S7 Fig for normalized counts of individual RNA targets). It is important to note that the increased brain content of phospholipids containing fatty acids derived from desaturase enzymes (Fig 2B and 2D) is consistent with increased expression of Srebp-1 target genes. Consistent with a previous report [6], Srebp-1c is the major Srebp-1 isoform in brain and up-regulated in brains of 2aECKO mice (S8 Fig). Next, we tested whether Srebp target genes would be up-regulated due to DHA deficiency prior to the development of microcephaly. Indeed, 2aECKO brains at e18.5 had a significant up-regulation of Srebp-1 and Srebp-2 target gene expression relative to littermate controls (Fig 3F). These findings indicate that up-regulation of Srebp target gene expression might be a proximal adaptation to DHA deficiency, and the first demonstration of increased Srebp target gene expression as a result of DHA deficiency in brain.

The rate-limiting step of de novo lipogenesis pathways involves the transport of precursor Srebp from the ER to the Golgi apparatus for proteolytic processing to release the nuclear form of Srebp [7–9]. Consistent with increased Srebp-1 target gene expression, western blot analysis on cerebrum lysates from 2aECKO P8 brains showed increased levels of the nuclear form of Srebp-1 (Fig 3G). Despite an increase in Srebp-2 target gene expression, increased nuclear Srebp-2 was not detected in 2aECKO brain. The level of Scd1, as a readout of Srebp-1 activity, was significantly increased in 2aECKO brains (Fig 3G). As Scd1 is a Srebp-1 target gene (and its up-regulation is consistent with increased Srebp-1 activity in Mfsd2a deficiency) and is an important, rate-limiting desaturase enzyme in fatty acid synthesis [31,32], it was subsequently used as a protein readout for Srebp regulation.

### Neural stem cells as an in vitro model for Mfsd2a and LPC-DHA function

In order to test the hypothesis that LPC-DHA transported by Mfsd2a suppresses Srebp activity, an in vitro cell model was needed that endogenously expresses Mfsd2a. We were unable to culture primary mouse brain endothelial cells that retained expression of Mfsd2a when plated in vitro (see Materials and methods for protocol that was used for primary cell isolation and culture). Immunolocalization of Mfsd2a in e18.5 WT and 2aKO brains showed that, in addition to Mfsd2a expression in the endothelium of the BBB, Mfsd2a was also found to be expressed in cells surrounding the ventricle, a region known to contain neural stem/progenitor cells (NSCs), and co-localized with the neural stem cell marker Nestin (S9A and S9B Fig) [33]. To determine if NSCs express Mfsd2a, we generated an NSC deficiency model of Mfsd2a (NSC^KO^) using a Nestin-cre driver line that is expressed in NSCs. The benefit of studying NSCs derived from NSC^KO^ mice is that these mice do not have reduced brain weight or DHA deficiency (S9C and S9D Fig, respectively), thus reducing potential confounding effects on NSC gene expression. Consistent with immunofluorescence data, western blot analysis showed Mfsd2a was expressed in NSC^WT^ derived from 2a^*fl/fl*^ mice but not in NSC^KO^ cells (S9E Fig). In order to determine the functionality of Mfsd2a in these cells, LPC-[^14^C]DHA transport assay was carried out. NSC^KO^ cells exhibited a significant 70% reduction in transport of LPC-[^14^C]DHA relative to NSC^WT^ cells, indicating that Mfsd2a expressed in NSCs is functional (S9F Fig).

### LPC-DHA represses de novo lipogenesis pathways

**Fig 4:**
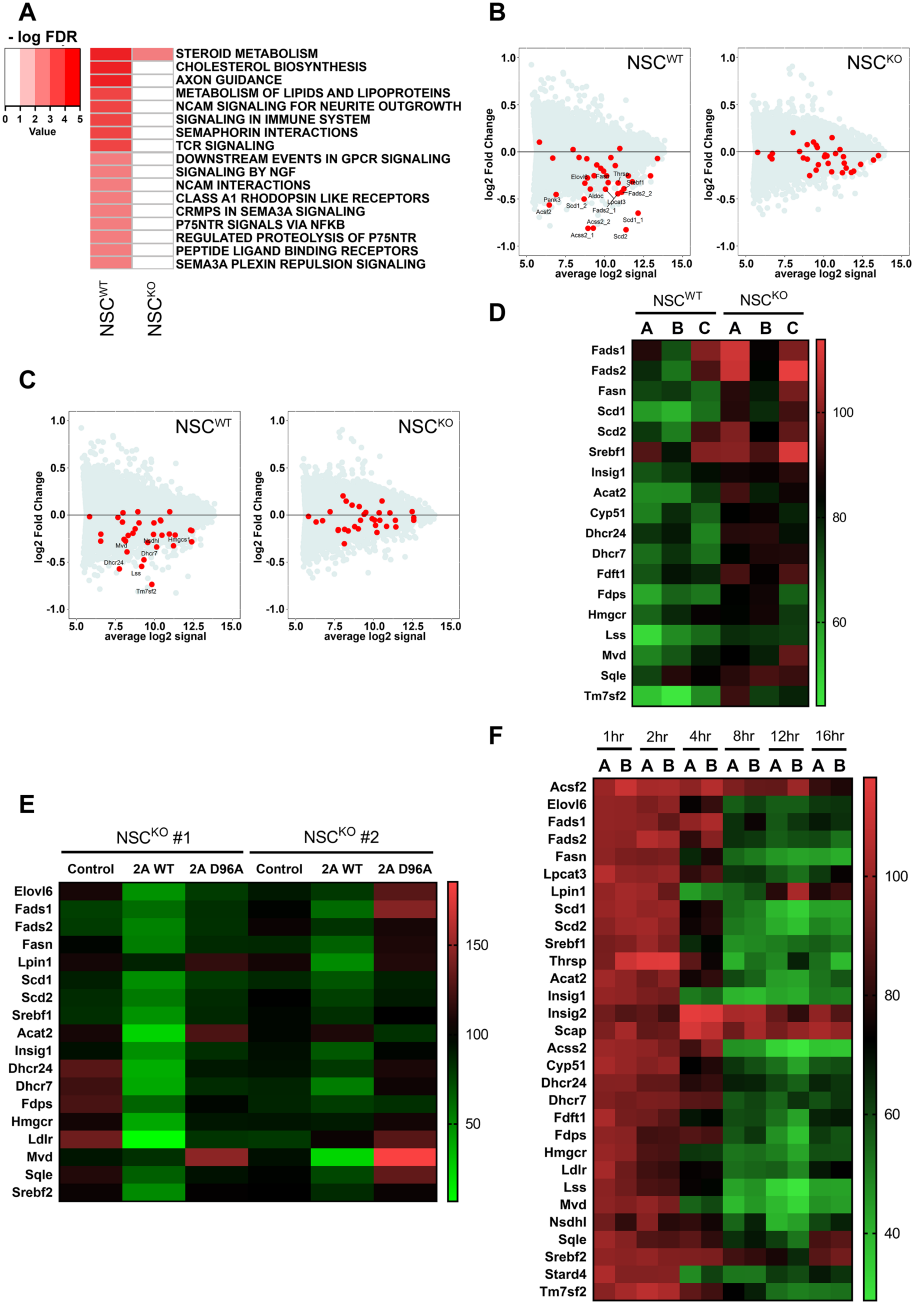
LPC-DHA represses de novo lipogenesis pathways. (A) Gene microarray analysis of NSC^WT^ and NSC^KO^ treated with or without 50 μM LPC-DHA. Heatmap represents commonly down-regulated Reactome pathways in response to LPC-DHA treatment relative to untreated control cells with an FDR ≤5%. RNA was pooled from 4–5 biological replicates according to their genotype and treatment condition. Color key indicates negative logarithm of the FDR for the selected pathways, for which a higher value indicates the pathway is more significantly different. (B) Srebp-1 target genes were down-regulated by LPC-DHA treatment. MA plot of gene microarray analysis of NSC^WT^ and NSC^KO^ treated with or without 50 μM LPC-DHA. Lipogenic targets of Srebp-1 were down-regulated in LPC-DHA–treated NSC^WT^ but not NSC^KO^ cells. Red dots represent individual Srebp-1 targets with significant changes over the background of expressed genes. (C) Srebp-2 target genes were down-regulated by LPC-DHA treatment. MA plot of gene microarray analysis of NSC^WT^ and NSC^KO^ treated with or without 50 μM LPC-DHA. Lipogenic targets of Srebp-2 were down-regulated in NSC^WT^ but not NSC^KO^ cells with LPC-DHA treatment. Red dots represent individual targets’ significant changes in this pathway over the background of expressed genes. (D) Confirmation of down-regulation of Srebp-1 and Srebp-2 targets using direct mRNA quantification by Nanostring analysis. Heatmap illustrates percentage of normalized counts of NSCs treated with LPC-DHA over untreated control. Down-regulation of Srebp targets were observed in NSC^WT^ with LPC-DHA treatment. Three biological replicates for each genotype were used and are indicated by capital letters above each lane. (E) Re-expression of WT Mfsd2a but not transporter inactive D96A mutant of Mfsd2a in NSC^KO^ cells restored sensitivity of cells to down-regulation of Srebp-1 and Srebp-2 pathways by LPC-DHA treatment. NSC^KO^ cells were transduced with Mfsd2a WT or Mfsd2a D96A adenovirus and treated with or without 50 μM LPC-DHA. Heatmap illustrates percentage of normalized counts NSCs treated with LPC-DHA over untreated control. Two biological replicates (NSC^KO^ #1 and NSC^KO^ #2) were used for each adenovirus transduction indicated above the lane in the heatmap. Control are cells not transduced with adenovirus. (F) Time course for down-regulation of Srebp-1 and Srebp-2 target genes in NSC^WT^ treated with or without 50 μM LPC-DHA over 1–16 hours. Heatmap illustrates percentage of normalized counts of NSC^WT^ treated with LPC-DHA over respective time point untreated control. Significant repression of Srebp pathways begin as early as 4 hours after LPC-DHA administration. Technical replicates were carried out for each time point. Numerical values underlying panels 4D–F can be found in S1 Data.

Activation of Srebp-1 and Srebp-2 target gene expression in brains of Mfsd2a deficiency models raised the hypothesis that LPC-DHA transported by Mfsd2a can repress lipogenesis pathways. To determine if LPC-DHA represses transcription of lipogenic genes, NSC^WT^ and NSC^KO^ cells were treated with or without 50 μM LPC-DHA for 16 hours, after which RNA was isolated for transcriptome analysis using gene microarray. The top predicted Reactome pathways that were down-regulated in response to LPC-DHA treatment in NSC^WT^ cells included lipogenic and cholesterogenic pathways (Fig 4A). LPC-DHA–treated NSC^KO^ cells did not exhibit significant down-regulation of these pathways, consistent with Mfsd2a-dependent transport of LPC-DHA (Fig 4A). MA plots of raw gene microarray data indicated that Srebp-1 and Srebp-2 target genes were down-regulated in NSC^WT^, for which red dots represent individual targets in the Srebp-1 (Fig 4B) and Srebp-2 (Fig 4C) pathway. These microarray findings were validated using Nanostring analysis (Fig 4D).

To further demonstrate that cell autonomous effects of down-regulation of Srebp-1 and Srebp-2 target gene expression by LPC-DHA are Mfsd2a dependent, we reintroduced WT Mfsd2a into NSC^KO^ cells using adenovirus transduction. As a control, NSC^KO^ cells were transduced with adenovirus expressing the Mfsd2a inactive mutant harboring a point mutant in the sodium-binding site where residue Aspartate-96 is converted to Alanine (Mfsd2a D96A) [23]. Non-adenovirus–transduced NSC^KO^ cells were used as an additional control. Western blot analysis confirmed Mfsd2a expression in NSC^KO^ cells transduced with Mfsd2a WT or Mfsd2a D96A (S10A Fig). Indeed, in LPC-DHA–treated NSC^KO^ cells re-expressing WT Mfsd2a, Srebp target genes were repressed relative to controls (Fig 4E). In contrast, repression of Srebp target genes was not observed in LPC-DHA–treated NSC^KO^ cells expressing Mfsd2a D96A mutant, consistent with repression of Srebp target genes being dependent on Mfsd2a transport activity (Fig 4E).

Inhibition of Srebp processing and therefore target gene expression in response to cholesterol loading or treatment of cells with 25-hydroxycholesterol occurs on a rapid timescale of several hours [8,34]. To determine the kinetics of LPC-DHA–mediated down-regulation of Srebp target gene expression, we quantified transcriptomic changes from 1 to 16 hours of exposure to LPC-DHA in NSC^WT^ cells using Nanostring analysis. Significant down-regulation of Srebp-1 and Srebp-2 target genes was observed as early as 4 hours posttreatment relative to untreated time point–matched control cells (Fig 4F). The inhibitory effect plateaued between 12 and 16 hours posttreatment. These findings indicate that down-regulation of Srebp-1 and Srebp-2 target gene expression in response to LPC-DHA treatment is rapid and exhibited a similar kinetic profile.

### LPC-DHA inhibits Srebp activity and de novo lipogenesis

**Fig 5:**
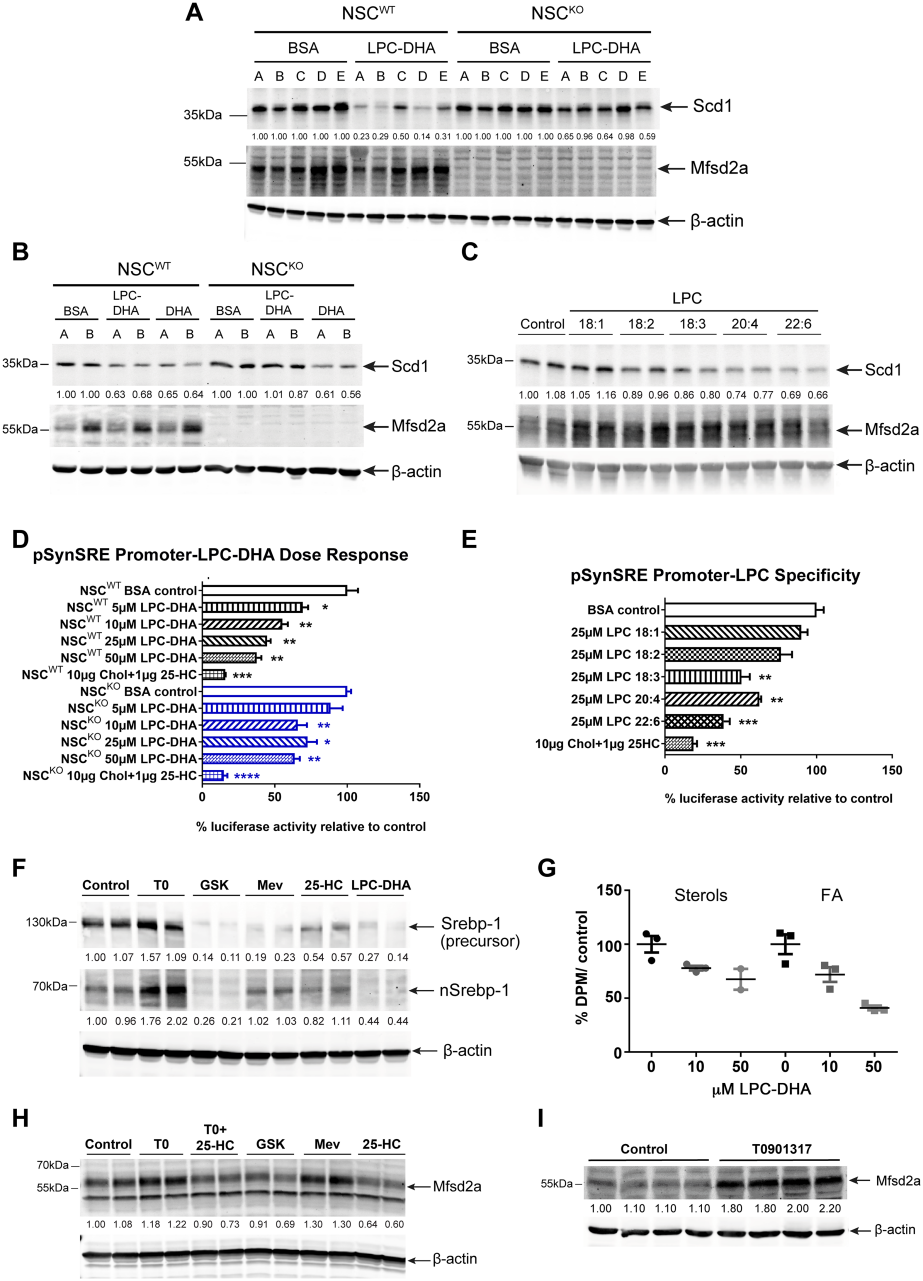
LPC-DHA inhibits Srebp activity and de novo lipogenesis and Mfsd2a is regulated by Srebp. (A) Western blot analysis of Scd1 and Mfsd2a expression in NSC^WT^ and NSC^KO^ treated with or without 50μM LPC-DHA. Scd1 expression in NSC^WT^ cells decreased with LPC-DHA treatment, but to a lesser extent in NSC^KO^ cells. β-actin served as a loading control. Capital letters above each lane represent biological replicates for each genotype (*n* = 5 for NSC^WT^ and NSC^KO^). (B) Western blot analysis of Scd1 and Mfsd2a expression in of NSC^WT^ and NSC^KO^ treated with or without 50 μM LPC-DHA or 50 μM DHA. DHA works in an Mfsd2a-independent manner to decrease Scd1 expression to a similar extent in NSC^WT^ and NSC^KO^ cells. β-actin served as a loading control. Capital letters above each lane represent biological replicates for each genotype (*n* = 2 for NSC^WT^ and NSC^KO^). (C) Specificity of LPC species for down-regulation of Scd1. Western blot analysis of Scd1 and Mfsd2a expression in NSC^WT^ treated with or without 50 μM each of LPC18:1, LPC18:2, LPC18:3, LPC20:4, or LPC22:6. Scd1 expression is more greatly reduced with increasing unsaturation of LPC-PUFAs. Mfsd2a expression remained relatively unchanged. β-actin served as a loading control. Technical replicates were carried out for each LPC treatment. (D) Dose-response effect of LPC-DHA on Srebp transcriptional activity. NSC^WT^ and NSC^KO^ cells were transfected with pSynSRE and treated with or without increasing concentrations of LPC-DHA, as indicated, or 25-hydroxycholesterol and cholesterol, as a positive control for repression of Srebp activity. The bar chart shows percent luciferase activity to respective untreated control as mean ± SE. LPC-DHA inhibited Srebp transcriptional activity in a concentration-dependent fashion in NSC^WT^ but not NSC^KO^ cells. Three technical replicates were carried out for each treatment condition. *p < 0.05; **p < 0.01, ***p < 0.001; ****p < 0.0001. (E) Specificity of LPC species for down-regulation of Srebp transcriptional activity. NSC^WT^ cells transfected with pSynSRE and treated with or without 25 μM each of LPC18:1, LPC18:2, LPC18:3, LPC20:4, or LPC22:6 or 25-hydroxycholesterol and cholesterol (sterols). The bar chart shows percent luciferase activity to untreated controls as mean ± SE. Sterols inhibit Srebp and were used as a positive control for inhibition of Srebp transcriptional activity. Increased repression of Srebp transcriptional activity relative to control was observed with increasing unsaturation of LPC-PUFAs, consistent with effects observed for down-regulation of Scd1 expression shown in panel (C). The bar chart shows percent luciferase activity to untreated control as mean ± SE. Three technical replicates were carried out for each treatment condition. **p < 0.01, ***p < 0.001. (F) LPC-DHA reduces nuclear Srebp-1 levels. Western blot analysis of Srebp-1 expression in NSC^WT^ treated with or without 50 μM of LPC-DHA. Activators and inhibitors of Srebp-1 processing and expression were used as controls to benchmark the effect of LPC-DHA. T0901317 (T0), an LXR agonist; GSK2033 (GSK), an LXR antagonist; Mevastatin plus mevalonic acid (Mev) inhibits HMG-CoAR activity; 25-hydroxycholesterol plus cholesterol (25-HC) inhibits Srebp-2 processing. β-actin served as a loading control. Technical replicates were carried out for each treatment condition. (G) LPC-DHA treatment reduced biosynthesis of sterols and fatty acids (FAs). [^14^C]-acetate lipogenesis assay was performed on NSC^WT^ cells. NSC^WT^ cells were treated with either 10 μM or 50 μM of LPC-DHA for 16 hours followed by supplementing cell media with [^14^C]-acetate for 2 hours. Sterols and fatty acids were purified as described in Materials and methods and quantified by scintillation counting. Uptake is expressed as mean percent DPM of untreated cells ± SE. Sterol synthesis was significantly reduced in 10 μM LPC-DHA–treated NSC^WT^ relative to control (*p < 0.05). Fatty acid synthesis was significantly reduced in 50 μM LPC-DHA–treated NSC^WT^ relative to control (**p < 0.01). Two to three technical replicates were carried out for each treatment condition. (H) Mfsd2a levels are regulated by Srebp activity. Western blot of Mfsd2a expression in NSC^WT^ cells treated with or without activators or inhibitors of Srebp-1 and Srebp-2, as described in (F) above. β-actin served as a loading control. Technical replicates were carried out for each treatment condition. (I) LXR agonist treatment of mice enhances brain levels of Mfsd2a. Western blot analysis of Mfsd2a expression in of brain lysates of WT mice injected with 30 mg/kg/day T0901317 or vehicle control for 4 days. β-actin served as a loading control. Control, *n* = 4; T0901317, *n* = 4; biological replicates. Experimental data depicted in this figure can be found in S1 Data.

To investigate the specificity for LPC species and Mfsd2a dependency for repression of Srebp target gene expression, we used Scd1 protein levels as an initial readout. Western blot analysis was carried out using lysates on individual NSC^WT^ and NSC^KO^ cell lines isolated from 5 different mice for each genotype. Indeed, Scd1 protein levels were significantly decreased in all 5 NSC^WT^ lines to a greater extent than in NSC^KO^ lines (Fig 5A). These findings indicate both robustness of the repressive effect and dependency on Mfsd2a transport. It is known that PUFAs, which can enter cells through diffusion and not via Mfsd2a [23], repress Srebp-1 processing [35]. Treatment of NSCs with unesterified DHA reduced Scd1 levels to a similar extent in both NSC^WT^ and NSC^KO^ cells, indicating that repression of Srebp activity in response to unesterified DHA is normal in NSC^KO^ cells (Fig 5B). Although transporter-independent passive diffusion of unesterified DHA occurs in vitro and represses Srebp-1 activation, previous findings have clearly demonstrated that brain uptake and accretion of DHA in vivo are dependent on LPC-DHA and Mfsd2a [23,36].

To explore the nature of LPC specificity for repression of Srebp pathway genes, NSC^WT^ cells were treated with species of LPC having fatty acids with varying degrees of unsaturation and carbon chain length. Western blot analysis indicated that Scd1 levels declined with increasing unsaturation of the LPC, in line with Mfsd2a having a higher affinity for transporting LPCs with polyunsaturated acyl chains like DHA [23] (Fig 5C). To corroborate these findings using a different approach, we first determined the dose-response effect of LPC-DHA on Srebp transcriptional activity. Measurement of transcriptional activity was carried out by utilizing an 3-hydroxy-3-methylglutaryl coenzyme A (HMG-CoA) synthase promoter (pSynSRE) plasmid construct containing 2 sterol response elements upstream of a luciferase reporter gene [37]. NSC^WT^ and NSC^KO^ cells transfected with pSynSRE were treated with increasing concentrations of LPC-DHA, and percent luciferase activity of control nontreated cells was quantified. Treatment of cells with 25-hydroxycholesterol plus cholesterol was used as a positive control for inhibition of Srebp transcriptional activity. LPC-DHA inhibited Srebp transcriptional activity in a concentration-dependent fashion in NSC^WT^ but not NSC^KO^ cells, with approximately 50% inhibition at 25 μM LPC-DHA (Fig 5D). This finding is significant because these effective LPC-DHA concentrations are within the physiological concentrations for LPCs containing polyunsaturated fatty acids (LPC-PUFA) found in human and mouse plasma [26,27,38]. Consistent with western blot data for Scd1 (Fig 5C), Srebp transcriptional activity was inhibited to a greater extent by LPCs with increasing unsaturation (Fig 5E).

The effect of LPC-DHA to inhibit Srebp transcriptional activity would strongly point to an inhibitory effect on Srebp processing. To test this idea, western blot analysis was carried out to detect Srebp-1 and Srebp-2 precursor and nuclear forms in NSC^WT^ cells treated with LPC-DHA. Positive controls for increased processing of Srebp-1 and Srebp-2 were the Liver X Receptor (LXR) agonist T0101317 and Mevastatin, respectively. LXR activation increases Srebp-1 expression and processing [39–41], while the LXR antagonist GSK2033 reduces Srebp-1 expression [42]. Positive controls for inhibition of Srebp-1 and Srebp-2 processing were the LXR antagonist GSK2033 and 25-hydroxycholesterol plus cholesterol, respectively. Treatment of NSC^WT^ cells with activators or inhibitors of Srebp-1 processing gave the expected outcomes, indicating that Srebp-1 in NSC^WT^ cells exhibited canonical regulation. LPC-DHA treatment was effective in significantly reducing both nuclear and precursor Srebp-1 in cells (Fig 5F), indicating that LPC-DHA acts at the level of Srebp-1 processing. However, because Srebp-1 regulates its own gene expression, we cannot be certain that the proximal effect of LPC-DHA is solely on Srebp-1 processing. In contrast, while positive and negative activators of Srebp-2 processing resulted in expected outcomes on Srebp-2 processing, LPC-DHA did not detectably reduce nuclear Srebp-2 (S10B Fig). Whether this finding indicates a novel mechanism whereby LPC-DHA regulates Srebp-2 transcriptional activity remains to be determined. Nonetheless, to determine if inhibition of Srebp-1 and Srebp-2 activity by LPC-DHA reduces fatty acid and sterol synthesis in NSCs, an in vitro [^14^C]-acetate lipogenesis assay was carried out. NSC^WT^ cells were treated with either 10 μM or 50 μM of LPC-DHA for 16 hours before supplementing the media with [^14^C]-acetate for 2 hours. Sterols and fatty acid fractionations were purified and radioactivity was quantified. Indeed, 50 μM LPC-DHA reduced the synthesis of cholesterol by 25% and fatty acids by 55% (Fig 5G), consistent with reduced Srebp activity and target gene expression.

### Mfsd2a is regulated by Srebp

Regulation of Srebp processing is feedback inhibited by products of Srebp target genes, raising the question of whether Mfsd2a expression is regulated by Srebp. To test this possibility, NSC^WT^ cells were treated similarly as in Fig 5F with activators and inhibitors of Srebp-1 and Srebp-2 processing. Interestingly, LXR agonist T0901317 and Mevastatin increased Mfsd2a expression (Fig 5H). Treatments with LXR antagonist GSK2033 had a minor effect on reducing basal Mfsd2a expression, while treatment with 25-hydroxycholesterol plus cholesterol had a large effect on reducing the expression of Mfsd2a (Fig 5H). A combination of T0901317, 25-hydroxycholesterol plus cholesterol treatment, which increases LXR and Srebp-1 activity but decreases Srebp-2 processing, mildly reduced Mfsd2a expression, similar to that of GSK2033 (Fig 5H). Consistent with LXR/Srebp-1 regulation of Mfsd2a in NSC cells, Mfsd2a protein levels were increased 2-fold in brains of T0901317–treated WT mice (Fig 5I). Importantly, data mining publicly available chromatin immunoprecipitation sequencing (ChIPseq) and gene array studies revealed Srebp-2 and LXRbeta binding sites in mouse Mfsd2a intron 3 using ChIPseq [43,44], and enhanced Mfsd2a expression in livers of mice treated with Lovastatin plus Ezetimibe [43] or in livers overexpressing Srebp but down-regulated in Scap-deficient livers [45]. Collectively, these data indicate that expression of Mfsd2a is regulated in part by LXR/Srebp-1 and Srebp-2.

### LPC-DHA affects membrane phospholipid saturation

**Fig 6:**
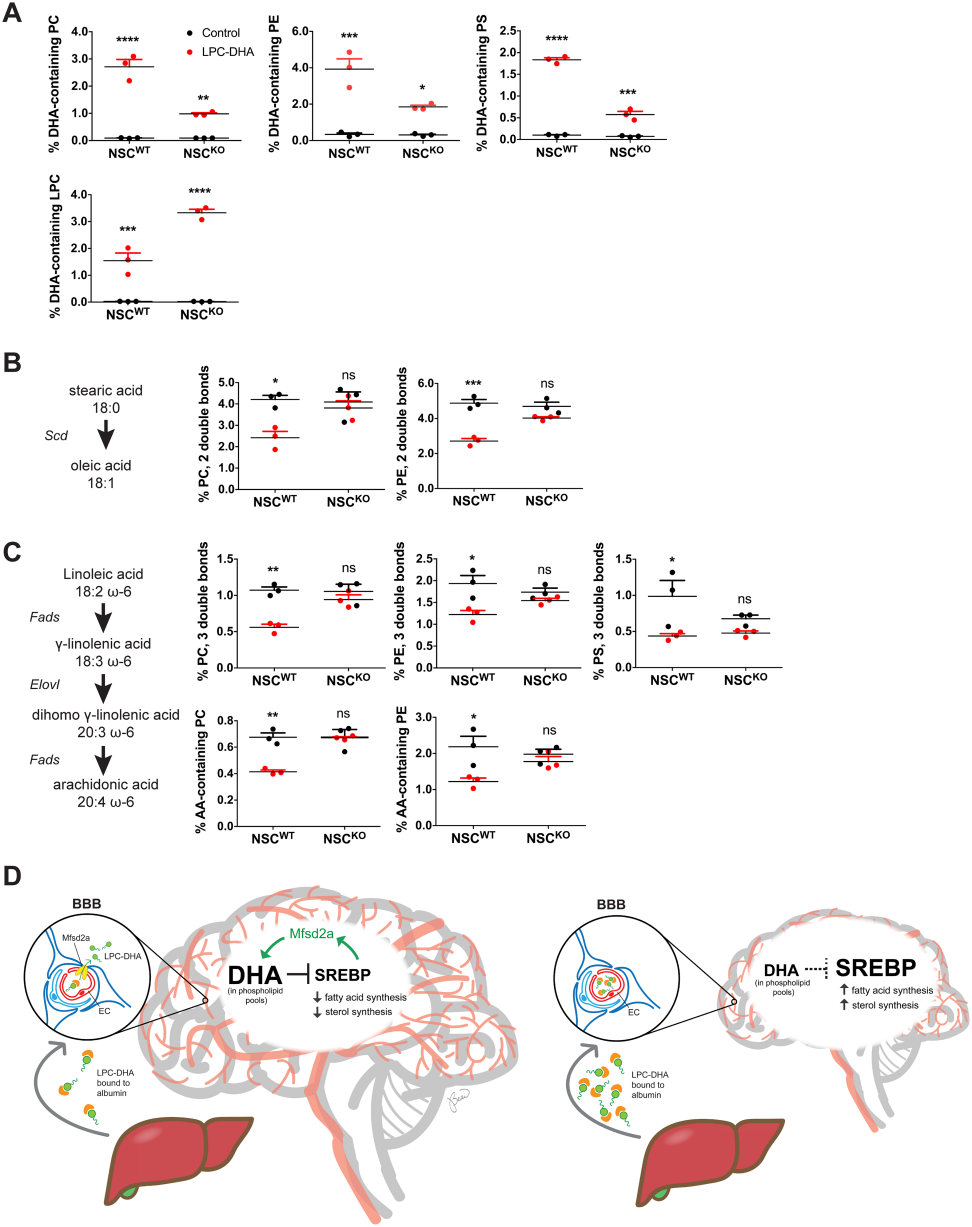
LPC-DHA affects membrane phospholipid saturation. Targeted lipidomic analysis of NSC^WT^ and NSC^KO^ cells treated with or without 50 μM LPC-DHA for 16 hours. (A) NSC^WT^ cells showed increased DHA in PC, PE, and PS relative to NSC^KO^ cells. Data are represented as percentage PC, PE, PS, and LPC containing DHA in phospholipids and represented as mean ± SE. (B) The biochemical pathway mediated by Scd1 is illustrated to highlight the lipid products quantified in this panel. Data are represented as percentage PC and PE phospholipids with fatty acid unsaturation of 2 double bonds and represented as mean ± SE. (C) The biochemical pathway mediated by Fads and Elovl enzymes is illustrated to highlight the lipid products quantified in this panel. Data are represented as percentage of PC, PE, and PS phospholipids containing a total fatty acid unsaturation of 3 double bonds and arachidonic acid (AA) and represented as mean ± SE. (D) Model for physiological role of LPC-DHA in regulating membrane phospholipid homeostasis and brain growth. In the developing brain, de novo lipogenesis mediated by Srebp-1 and Srebp-2 and accretion of DHA in the form of lysophosphatidylcholine (LPC-DHA) uptake at the BBB by Mfsd2a are essential for brain development. LPCs are synthesized by the liver and circulate in blood bound to albumin. In the left panel, Mfsd2a is present at the BBB to transport LPC-DHA from blood into brain, resulting in enrichment of DHA in phospholipid pools during pre- and postnatal brain development. Phospholipid DHA pools attenuate Srebp activity, leading to decreased lipogenesis. Moreover, Mfsd2a itself is regulated by Srebp, forming a negative feedback loop to balance de novo lipogenesis with exogenous uptake of LPC-DHA, with the result of maintaining homeostasis of membrane phospholipid composition. Conversely, in the right panel, Mfsd2a deficiency at the BBB, consistent with the lack of LPC-DHA transport across the BBB into brain, LPC-DHA accumulates in plasma and postnatal brain growth and phospholipid DHA pools are reduced, resulting in microcephaly. Consequential to reduced levels of phospholipid DHA pools prior to the onset of microcephaly, Srebp activity is enhanced, leading to a compensatory increase in lipogenesis. For panels (A–C), 3 biological replicates of NSC^WT^ control, NSC^WT^ plus LPC-DHA, NSC^KO^ control, and NSC^KO^ plus LPC-DHA were used. ****p < 0.0001; ***p < 0.0002; **p = 0.0021; *p = 0.0332. Numerical values underlying panels 6A–C can be found in S1 Data.

Given our findings that LPC-DHA negatively regulates Srebp activity in an Mfsd2a-dependent fashion, we addressed whether these effects translated into significant changes in membrane lipid composition. To test this concept, NSC^WT^ and NSC^KO^ cells were treated with LPC-DHA for 16 hours followed by targeted lipidomic analysis. As anticipated, NSC^WT^ cells were significantly enriched in DHA-containing PC, PE, and PS relative to NSC^KO^ cells, consistent with transporter-mediated uptake of LPC-DHA (Fig 6A, heatmaps illustrating percentage of individual PC, PE, and PS phospholipid species shown in S11 Fig). In contrast, NSC^KO^ cells had higher levels of LPC-DHA than NSC^WT^ cells, which is also consistent with reduced LPC-DHA uptake and bioconversion into PC, PE, and PS phospholipid classes relative to NSC^WT^ cells (Fig 6A).

Remarkably, LPC-DHA–treated NSC^WT^ cells, but not NSC^KO^ cells, had a significant reduction in PC and PE phospholipids with fatty acid sum composition containing 2 double bonds (Fig 6B and S11 Fig), consistent with reduced expression of the rate-limiting enzyme Scd (Fig 5A). Moreover, LPC-DHA treatment of NSC^WT^ cells resulted in significantly reduced PC, PE, and PS phospholipids with fatty acid sum composition containing 3 double bonds (Fig 6C and S11 Fig) and AA (Fig 6C and S11 Fig), consistent with repression of gene expression of desaturase and elongase enzymes such as Fads and Elovl (Fig 4F). Taken together, these findings indicate that LPC-DHA treatment at physiological levels has a profound effect on membrane lipid composition over a relatively short time frame and furthermore indicate that the phospholipid pool containing unsaturated fatty acids is highly dynamic through modulation of Srebp activity by LPC-DHA.

## Discussion

DHA, an essential and abundant lipid in brain thought to be critical for normal brain development, is transported into brain as LPC by Mfsd2a. However, biochemical functions of DHA in brain that make it essential are not well understood. In the current study, we have addressed several fundamental questions pertaining to the role of LPC-DHA and Mfsd2a in brain growth and determined a critical role for DHA in regulating membrane lipid composition through regulation of lipogenic pathways. Our findings support the following 2 major conclusions: (1) Mfsd2a expression is required at the BBB for normal postnatal brain growth and Mfsd2a deficiency activates Srebp-1 and Srebp-2 target gene expression; (2) LPC-DHA transported by Mfsd2a represses Srebp activity, leading to reduced de novo lipogenesis and synthesis of PUFAs. These combined findings indicate that Mfsd2a–mediated transport of LPC-DHA has profound effects on membrane phospholipid composition during brain development, in part through its regulation of the Srebp pathway.

Most genetic forms of microcephaly are categorized as primary microcephaly disorders, which result from defects in cellular proliferation and differentiation during prenatal brain development [30], while postnatal forms of microcephaly manifest as defects in neuron arborization [46]. Using an inducible endothelial-specific Mfsd2a deletion mouse model, we have definitively proven that Mfsd2a deficiency results in a unique form of microcephaly manifesting as postnatal microcephaly. Similar to other genetic causes of postnatal microcephaly, such as Angelman Syndrome, Rett Syndrome, and Christianson Syndrome [46], postnatal neurons of Mfsd2a deficiency mouse models fail to fully arborize, a process that relies on lipogenesis [47,48]. Importantly, similar to our findings in mice, humans with loss-of-function mutations in Mfsd2a are born with head circumferences in the normal or slightly reduced range, and develop severe microcephaly within the first year of life [27]. It has been reported that 2aKO mice have increased BBB permeability through the process of transcytosis, which is normally absent in the endothelium of the BBB [49], raising the possibility that a leaky BBB might be causative for microcephaly and DHA deficiency in this model. However, it has been reported that BBB permeability, but not microcephaly or DHA deficiency, can be completely rescued in 2aKO mice by genetic deficiency of Caveolin-1 (Cav-1). Moreover, mice with a homozygous mutation of the sodium-binding residue Aspartate-96 to Alanine-96 in Mfsd2a, a mutation that completely inactivates lipid transport function but does not affect expression, also presented with microcephaly and DHA deficiency [25]. These findings indicate that microcephaly and DHA deficiency are primary phenotypes of Mfsd2a deficiency and not a result of a leaky BBB [25], and LPC transport activity is required for DHA accretion and brain growth.

Our findings revealed that DHA deficiency and activation of the Srebp-1 and Srebp-2 pathways are present prenatally before the onset of microcephaly. This is the first evidence of regulation of Srebp-1 and -2 in brain in response to changing DHA levels. What would be the biochemical function of regulating Srebp activity in response to the uptake of LPC-DHA and accretion of DHA into phospholipid pools during the rapid stages of brain growth? A likely explanation based on our findings points to issues of not only membrane biogenesis but membrane composition, in which a tight homeostatic balance might be predicted to be achieved between cholesterol and phospholipid-containing PUFA, with each having opposite effects on membrane fluidity and function [50]. Because LPC-DHA uptake and thus membrane phospholipid levels of DHA might be regulated by a homeostatic mechanism similar to regulation of cholesterol synthesis and turnover in the brain, the effects that we report here for LPC-DHA on membrane composition might be most relevant to the growing brain, or in aging or neurological disease in which brain DHA levels decline and Srebp lipogenic target genes have been reported to be elevated [11,51–55].

It is understood that the PUFAs AA and DHA down-regulate Srebp-1, but not Srebp-2, processing and activation of target gene expression in both immortalized cell lines in culture and in mouse liver [35,56–61]. The characterized biochemical mechanisms for PUFA effects on inhibiting Srebp-1 processing involve both direct effects on the ER-associated degradation machinery mediating the degradation of Insig-1, and inhibition of LXR activity that regulates Srebp-1 expression and processing [35,57,62]. There is recent evidence that oxysterols generated from products of the Srebp-2 pathway and acting through LXR are required to maintain Srebp-1 expression in liver [63,64]. An intriguing aspect of our findings is that Srebp-1 and Srebp-2 target genes were reduced on a similar timescale following LPC-DHA treatment in NSCs, but without detectable reductions in the nuclear form of Srebp-2. These data might imply the existence of a mechanism that links Srebp-1 with Srebp-2 activity to regulate cholesterol biosynthesis and membrane phospholipid saturation. In support of this idea, intestinal deletion of lysophosphatidylcholine acyltransferase 3 (LPCAT3), an important acyltransferase that generates polyunsaturated phospholipids [65–67], in mice resulted in elevated Srebp-2 activity and cholesterol synthesis, indicating that membrane phospholipid saturation can regulate membrane cholesterol biosynthesis via Srebp-2 [68].

Our combined findings are particularly relevant to the period in fetal and postnatal brain development involving a rapid and extensive acquisition of DHA and membrane lipid. We propose a model (Fig 6D) in which LPC-DHA transported via Mfsd2a at the BBB regulates the level of Srebp-1 and Srebp-2 activity, thereby balancing the need for de novo synthesis of membrane PUFA with exogenous PUFA uptake and impacting dynamic changes in membrane saturation and fluidity. Moreover, Mfsd2a is regulated by Srebp and constitutes a previously unknown negative feedback loop to modulate de novo lipogenesis.

## Materials and methods

### Ethics statement

All experimental protocols were approved by SingHealth Institutional Animal Care and Use Committee (IACUC protocol number #2015/SHS/1028). Anesthesia of mice was carried out using isoflurane at 2% mixed with oxygen. Mice that need to be perfused with PBS and/or 4% paraformaldehyde (PFA) prior to tissue harvest were anesthetized with a combination of ketamine (20 mg/kg body weight) and xylazine (2 mg/kg body weight).

### Mouse models

Mfsd2a whole-body KO (2aKO) mice were generated as described previously [69]. Mfsd2a floxed mice (2a^*fl/fl*^) and Mfsd2a endothelial-specific knockout (2aECKO) were generated as described previously [24]. Tamoxifen-inducible endothelial-specific knockout mice (2aiECKO) were generated by crossing 2a^*fl/fl*^ with Cdh5-cre/ERT2 driver [C57BL/6-Tg(Cdh5-cre/ERT2)1Rha [70]. To create an early postnatal Mfsd2a BBB-specific knockout, P0 pups were injected intraperitoneally for 3 consecutive days with 50 μg/g body weight 4-hydroxytamoxifen (Sigma-Aldrich, St. Louis, MO) dissolved in corn oil (injection scheme, S2A Fig). To generate NSC deficiency model of Mfsd2a (NSC^KO^), 2a^*fl/fl*^ mice were crossed to a Nestin-cre driver line (B6.Cg-Tg(Nes-cre)1Kln/J, the Jackson Laboratory). All mice were housed in colony cages on a 12-hour light/12-hour dark cycle with controlled humidity and temperature at 23 °C. WT and 2aKO mice were fed ad libitum with a high-energy diet 5LJ5 (LabDiet, St. Louis, MO) with a total of 11% fat, while all other animals were fed ad libitum on a normal chow diet (Global 18% Protein Rodent Diet from Harlan, Envigo, Madison, WI) and have free access to water. Pups were weaned at 3 weeks of age. Both male and female mice were used in all experiments and no gender differences were observed. Mice were transcardially perfused with PBS before cerebrums were extracted and snap-frozen in liquid nitrogen before downstream immunoblot, lipidomic, or transcriptomic analysis.

### Primary cell culture

Primary mouse brain vascular endothelial cells were isolated and cultured according to Navone and colleagues [71]. An NSC deficiency model of Mfsd2a was generated using a floxed allele of Mfsd2a (2a^*fl/fl*^) crossed to NSCs-cre driver Nestin (2aNKO). Mouse NSCs were isolated from 2a^*fl/fl*^ and 2aNKO from P5 neonates according to Chen and colleagues [72], with minor modifications. Both genders were used and every NSC line was derived from a single neonate. Briefly, hippocampi and cortices from each neonate were isolated in ice-cold HBSS (calcium- and magnesium-free) (Invitrogen/Thermo Fisher Scientific, Waltham, MA) and digested in 0.25% trypsin (Gibco/Thermo Fisher Scientific, Carlsbad, CA) for 30 minutes at 37 °C with constant agitation. After 3 washes with HBSS, tissues were triturated 10 times to obtain single cell suspension. Cells were pelleted at 500*g* and resuspended in Serum-Free Media supplemented with 20 ng/mL EGF (Stemcell Technologies, Vancouver, Canada) and 20 ng/mL FGF (Stemcell Technologies, Vancouver, Canada). Cells were cultured in a T75 flask at a density of 1×10^5^ cells/mL in incubators at 37 °C with 5% CO_2_. Media was changed the next day and cells were allowed to proliferate to form neurospheres. After 5–6 days in culture, when neurospheres reach 0.1 mm in diameter, NSCs were plated as a monolayer culture on 2% Matrigel (Corning, New York, NY) coated surface. NSCs were grown as neurospheres for maintenance and propagation while all NSC experiments in this study were performed as a monolayer culture.

### Cell treatment

NSCs were plated at the density of 1×10^5^ cells/mL on 2% Matrigel coated surface. Cells were allowed to proliferate and reach 80%–90% confluency before treatments were carried out. Overnight treatments (16 hours) were applied unless indicated otherwise. Twelve percent fatty acid–free BSA (Sigma-Aldrich, St. Louis, MO) was used as carriers for all lipids used for cell culture treatment. All control wells were treated with 12% BSA. Other compounds and final concentration used in this study are as follows: T0901317 (1 μM, Sigma-Aldrich, St. Louis, MO), GSK2033 (10 μM, Tocris, Bristol, UK), Mevastatin (50 μM, Sigma-Aldrich, St. Louis, MO), mevalonate (50 μM, Sigma-Aldrich, St. Louis, MO), 25-HC (1 μg/mL, Sigma-Aldrich, St. Louis, MO), and Cholesterol (10 μg/mL, Sigma-Aldrich, St. Louis, MO).

### RNA isolation

For RNA isolation from NSCs, cells were lysed in Buffer RLT from RNeasy Micro Kit (Qiagen, Hilden, Germany) and total RNAs were purified according to manufacturer’s recommendation. RNA concentration was quantified using Nanodrop (Thermo Fisher Scientific, Waltham, MA). For RNA isolation from half cerebrums, tissues were lysed in TRIzol (Thermo Fisher Scientific, Waltham, MA) using a MagNA Lyser Instrument (Roche, Basel, Switzerland). Total RNAs were extracted according to the protocol described by Untergasser Lab (Untergasser A. “RNAprep—TRIzol combined with Columns” *Untergasser’s Lab*. Winter 2008.). RNA concentration was quantified using Nanodrop.

### Protein analysis

For brain lysates, brain tissues were dounce homogenized in ice-cold RIPA (Thermo Fisher Scientific, Waltham, MA) buffer supplemented with EDTA-free protease and phosphatase inhibitors (Roche, Basel, Switzerland) and allowed to rotate at 4 °C for an hour. Supernatant was collected after 10 minutes centrifugation at maximum speed at 4 °C for western blot. For whole cell lysates, cells were lysed with RIPA buffer supplemented with EDTA-free protease and phosphatase inhibitors and allowed to rotate at 4 °C for an hour. Lysates were spun down for 10 minutes at 14,000*g* at 4 °C and supernatants were collected for western blot. For preparation of nuclear and cytoplasmic/membrane fractions from whole cell lysates, fractionation was carried out using NE-PER Nuclear and Cytoplasmic Extraction Kit (Thermo Fisher Scientific, Waltham, MA) according to the manufacturer’s recommendation. Preparation of cell lysates in Fig 5F and 5H, and S10B Fig were done according to [35], with modifications. NSCs were incubated with Calpain Inhibitor I (ALLN) (25 μg/mL, Sigma-Aldrich, St. Louis, MO) for 2 hours before cells were harvested by scraping in RIPA (Thermo Fisher Scientific, Waltham, MA) containing protease inhibitors aprotinin (2.8 μg/mL, Sigma-Aldrich, St. Louis, MO), ALLN (25 μg/mL, Sigma-Aldrich, St. Louis, MO), leupeptin (10 μg/mL, Sigma-Aldrich, St. Louis, MO), Pefabloc (0.5 mM, Sigma-Aldrich, St. Louis, MO), and pepstatin A (5 μg/mL, Sigma-Aldrich, St. Louis, MO). Protein concentration was quantified using BCA assay kit (Thermo Fisher Scientific, Waltham, MA). A total of 30 μg of cell lysates or 80 μg of brain lysates was resolved on SDS-PAGE, transferred to nitrocellulose membrane, and blocked in 5% nonfat milk (Bio-Rad Laboratories, Hercules, CA). Primary antibodies were diluted in 3% BSA containing 15 mM Sodium azide in TBS-T (50 mM Tris, 150 mM NaCl, 0.1% Tween-20) and incubated with the membrane at 4 °C overnight. After 3 washes with TBS-T, membranes were incubated with IR Dye-labeled secondary antibodies (LI-COR, Lincoln, NE) diluted in 3% BSA. Immunoblot signals were captured by ODYSSEY infrared imaging system (LI-COR, Lincoln, NE).

### Postnatal mice brain immunohistochemistry

To prepare brain sections, deeply anesthetized mice were transcardially perfused with PBS followed by 4% PFA in PBS. Brains were extracted and postfixed in PFA at 4 °C before samples were cryoprotected in 30% Sucrose in PBS overnight. Brains were embedded in OCT (Sakura Finetek USA, Torrance, CA) and coronal sections (20 μm) were obtained using Leica Cryostat CM1520. After heat antigen retrieval using sodium citrate buffer, brain sections were incubated with blocking buffer (10% Normal Goat Serum, 1% BSA, and 3 M Glycine in TBS-0.1% Triton-X) for 1 hour at room temperature (RT). After blocking, antibodies were diluted in blocking buffer and incubated overnight at 4 °C. The following antibodies were used: Anti-Cux1 (1:100, Santa Cruz, Dallas, TX), Ctip2 (1:100, Abcam, Cambridge, UK), Mfsd2a (1:100, in house), and Glut1 (1:200, Abcam, Cambridge, UK). After washing with TBS-T, brain sections were incubated with Alexa Fluor secondary antibodies (1:500, Invitrogen/Thermo Fisher Scientific, Carlsbad, CA). Nuclei were stained with Hoechst 33342 (Thermo Fisher Scientific, Waltham, MA) for 5 minutes before mounting with FluorSave Reagent (Merck Millipore, Burlington, MA). Images were obtained using LSM710 Confocal Microscope (Carl Zeiss, Oberkochen, Germany).

### Embryonic mice brain extraction for RNA or immunohistochemistry

Pregnant mice were humanely killed with CO_2_ and e18.5 embryos were extracted. Brains were dissected under stereomicroscope and snap-frozen in liquid nitrogen before processing for RNA extraction. To prepare brain sections for immunohistochemistry (IHC), dissected brains were rinsed in PBS before fixing in 4% PFA in PBS overnight at 4 °C. Brains were cryoprotected in 30% sucrose in PBS overnight before embedding in OCT. Coronal sections (20 μm) were obtained using Leica Cryostat CM1520. IHC was carried out similarly to that in adult brains. The following antibodies were used: Anti-Tbr1 (1:100, Abcam, Cambridge, UK), Ctip2 (1:100, Abcam, Cambridge, UK), Mfsd2a (1:100, in house), Glut1 (1:200, Abcam, Cambridge, UK), and Nes (1:100, Abcam, Cambridge, UK). Images were obtained using LSM710 Confocal Microscope (Carl Zeiss, Oberkochen, Germany).

### Gene expression analysis using microarray analysis

Equal amounts of RNA from 5–6 half cerebrums or 4–5 NSCs were pooled according to their genotype and treatment condition, and RNA integrity was verified using Bioanalyzer (Agilent Technologies, Santa Clara, CA). Microarray profiling was done on pooled samples with an RNA integrity cutoff of 8.5 using Mouse 430 2.0 arrays (Affymetrix/Thermo Fisher Scientific, Santa Clara, CA). Background-adjusted, quantile-normalized gene expression signals were generated via robust multichip averaging (RMA) method via the “affy” package in R. Filtered gene lists (genes with maximum expression in 2aKO or WT, 2a^*fl/fl*^ or 2aECKO, 2aiECKO with ≥50 in 2aKO, 2aECKO or 2aiECKO and fold-change ≥1.25; NSC^WT^ or NSC^KO^ with or without LPC-DHA treatment, ≥50 in BSA control NSC^WT^ or NSC^KO^ with fold-change ≤0.75) were analyzed using Gene Set Enrichment Analysis and Reactome pathway analysis to identify significantly altered canonical pathways commonly up-regulated with Mfsd2a deficiency or down-regulated with LPC-DHA treatment. Target validation was carried out by Nanostring analysis. Microarray data are available on GeoProfiles (www.ncbi.nlm.nih.gov), accession number GSE115971.

### Nanostring analysis (nCounter gene expression assay, Nanostring Technologies)

Custom gene expression CodeSets were designed based on gene targets of lipogenesis (Srebp-1) and cholesterogenesis (Srebp-2) pathways. Housekeeping genes were also included as a control. Pooled RNA samples that were used for microarray analysis were analyzed individually as biological replicates following the manufacturer’s instructions. Briefly, a master mix was first prepared by diluting the Reporter CodeSet with hybridization buffer. A total of 100 ng RNA was then hybridized with the Reporter CodeSet and Capture ProbeSet at 65 °C for 24 hours. Following the hybridization reaction, samples were loaded onto nCounter SPRINT cartridges and allowed to run on the nCounter SPRINT Profiler. Samples that meet the quality control requirements were normalized on nSolver Analysis Software 3.0. Heatmaps were also generated on nSolver Analysis Software 3.0.

### Luciferase assay

NSCs were cultured to 90% confluence on 24-well plates coated with 2% Matrigel. Each well was transfected with 0.5 μg pSynSRE [37] and 10 ng pRL Renilla luciferase with Lipofectamine LTX (Invitrogen/Thermo Fisher Scientific, Carlsbad, CA). Six hours after transfection, media were replaced and cells were treated with 12% BSA, 5–50 μM of LPC-DHA, or 25 μM of LPC-PUFAs of varying degrees of unsaturation and carbon length overnight. Cells were harvested with Dual-Luciferase Reporter Assay System (Promega, Madison, WI) according to the manufacturer’s instruction. Luminescence reading was quantified using Tecan Infinite M200 Microplate Reader. Relative luciferase activity was expressed as firefly luminescence/renilla luminescence ratio.

### In vitro lipogenesis assay

Twelve percent BSA, 10 μM LPC-DHA, or 50 μM LPC-DHA were added to 3 wells, respectively, for 16 hours. Following that, 2 μCi of [^14^C]Acetic acid (American Radiolabeled Chemicals, St. Louis, MO) was added to each well for 2 hours and lipids were extracted from the cells by 0.8 mL of Hexane:Isopropanol (3:2) solvent twice for each well. After solvent was evaporated under a steady stream of nitrogen gas, long-chain fatty acids and sterols were extracted from each well, as described previously [73]. Briefly, lipids were saponified with 5 N NaOH; sterols in the non-saponifiable portion were extracted with petroleum ether and precipitated with 0.5% digitonin (Wako) in 50% ethanol. The remaining portion, which contained long-chain fatty acids, was extracted by petroleum ether after reacidification with 10 N H_2_SO_4_. [^14^C] radioactivity was measured with Beckman Scintillation Counter.

### Lipidomic analysis

For the preparation of brain tissues for lipidomic analysis, frozen brain tissues were first lyophilized until constant weight was achieved. Phosphate buffered saline (PBS) was added to each sample in proportion to its respective dry weight (30 μL per mg) and homogenized using an Omni beadruptor homogenizer (speed: 5.45 meters/second; cycle: 8 times; duration: 45 seconds, dwell time: 10 seconds). Lipid extraction from homogenates was done according to the Bligh and Dyer method [74]. Briefly, 180 μL of freshly prepared, chilled extraction solvents (chloroform (CHCl_3_)/methanol (MeOH) 1/2, v/v) containing internal standards were added to 10 μL of homogenate. Samples were vortexed and then incubated with agitation on a thermomixer at a speed of 700 rpm at 4 °C in the dark for 1 hour. Organic phases were transferred to new tubes and the homogenates were re-extracted with 500 μL of chilled CHCl_3_. The organic phases were pooled together and dried in a speed vac. Dried lipid extracts were stored in −80 °C until they were ready for mass spectrometric (MS) analysis, and dissolved in 100 μL of CHCl_3_/MeOH 1:1 (v/v) prior to MS analysis. For the preparation of NSCs for lipidomic analysis, lipids were extracted using a hexane:isopropanol (3:2, v/v) solvent and dried down under a Nitrogen stream. Dried lipid extracts were stored in −80 °C until ready for MS analysis and were dissolved in 60 μL of CHCl_3_/MeOH 1:1 (v/v) containing internal standards prior to MS analysis. Samples were randomized for injection into a liquid chromatography-tandem mass spectrometry (LC-MS/MS) instrument (1290 Liquid Chromatography System, and 6460 QqQ, Agilent Technologies). Quality controls and blanks were injected after every 6 sample injections to monitor stability of the instrument response and carryover. The chromatographic column was a Kinetex HILIC (150×2.1 mm, 2.6 μM, 100 Å; Phenomenex). Gradient elution was undertaken with solvents A (50% acetonitrile/50% 25 mM ammonium formate buffer, pH 4.6) and B (95% acetonitrile/5% 25 mM ammonium formate buffer, pH 4.6), with a gradient range from 75% to 25% solvent B in 6 minutes, 90% to 10% solvent B in 1 minute, and 0.1% to 99.9% solvent B in 0.1 minute, followed by 0.1% to 99.9% solvent B for 3 minutes (total run time of 10.1 minutes). Phospholipids were quantified at the sum composition level using multiple reaction monitoring (MRM) with precursor to headgroup transitions. For lipidomics analysis of brains in Fig 2B and 2D, and S9D Fig, phospholipid with fatty acid sum composition containing 4 double bonds were considered AA containing, while phospholipid with a fatty acid sum composition containing 6 double bonds were considered DHA containing. For lipidomics analysis of NSC^WT^ and NSC^KO^ cells treated with or without 50 μM LPC-DHA in Fig 6A and 6C, phospholipid with fatty acid sum composition containing 4 double bonds were considered AA containing, while phospholipid with a fatty acid sum composition containing 6, 7, and 8 double bonds were considered DHA containing. MS parameters were gas temperature of 300 °C, gas flow of 5 L/minute, sheath gas flow of 11 L/minute, and capillary voltage of 3,500 V. Quantification data were extracted using MassHunter Quantitative Analysis (QQQ) software, and data were manually curated to ensure correct peak integration. Areas under the curve for the extracted ion chromatogram peaks for each MRM transition and lipid species were normalized to the internal standard. Isotope correction was then done on all relevant lipid species using an in-house R script.

### LPC-PUFA preparation

To prepare LPC-PUFAs, PC containing di-18:2, di-18:3, di-20:4, or di-22:6 in chloroform (Avanti Polar Lipids, Alabaster, AL) were used as a starting material. Chloroform was allowed to evaporate under a constant stream of nitrogen gas, and dried lipid films were redissolved in 95% ethanol. Following addition of Novozyme 435 (Sigma-Aldrich, St. Louis, MO), digestion was carried out at 55 °C for 36 hours with constant agitation. Digested lipids, which contain a mix of LPCs, free fatty acids, and undigested PCs, were extracted with chloroform:ethanol and allowed to resolve by thin-layer chromatography (TLC) with known lipid standards using a chloroform:ethanol:H_2_O:Triethylamine (30:35:7:35) solvent system. Lipid species on TLC plates were visualized by iodine exposure, and LPC-PUFAs were scraped off and extracted from silica with chloroform:methanol:H_2_O (5:5:1). Following a brief centrifugation step, the organic phase was allowed to evaporate under a constant stream of nitrogen gas. The total amount of LPC-PUFAs was quantified by LC/MSMS analysis. Dried lipid films were dissolved in 12% BSA to a final concentration of 10 mM.

### LPC-DHA transport assay

[^14^C]LPC-DHA (American Radiolabeled Chemicals, St. Louis, MO) was prepared as previously described [23]. NSCs were incubated with 50 μM [^14^C]LPC-DHA for 30 minutes at 37 °C with 5% CO_2_. Media were removed and cell layer was washed 3 times with PBS with 0.6% BSA. Cells were lysed in RIPA buffer and radioactivity was measured on the Beckman Scintillation Counter. [^14^C]LPC-DHA uptake activity was expressed as DPM per well.

### Mfsd2a complementation in NSC^KO^

Adenoviral vectors were obtained from ViraQuest. VQAd LSL Mfsd2a (6.0×10^10^ PFU/mL) carries WT mouse Mfsd2a and VQAd LSL D96A (5.0×10^10^ PFU/mL) carries mouse Mfsd2a D96A mutant, both under the control of CMV promoter (pVQAd CMV K-NpA). NSC^KO^ cells in monolayer were transduced overnight with Adenoviral vectors at a specific multiplicity of infection (MOI). Twelve percent BSA or 50 μM LPC-DHA was added to the NSC for another 16 hours. RNAs were extracted for Nanostring analysis.

### Quantitative reverse transcriptase-PCR

cDNA was synthesized from 1 μg of total RNA from cerebrums of P8 2a^*fl/fl*^ and 2aECKO mice using iScript cDNA Synthesis Kit (Bio-Rad Laboratories, Hercules, CA). Primer sequences for Srebp-1a and Srebp-1c were taken from Yang and colleagues [75]. Quantitative reverse transcriptase-PCR (qRT-PCR) was carried out using the SensiFAST SYBR Hi-Rox Kit (Bioline Reagents, London, UK). All reactions were carried out in triplicates.

### Golgi staining and Sholl image analysis

Golgi staining was carried out per the manufacturer’s instructions, with modifications. Briefly, anesthetized animals were perfused with PBS. Brains were extracted carefully using plastic tools and immediately immersed in impregnation solution protected from light and stored at RT. Impregnation solution was prepared at least 24 hours in advance by mixing equal volumes of Solution A and Solution B. Extra care was taken to avoid agitation of the brain samples. The impregnation solution was replaced the next day and brain samples were left undisturbed for the next 48 hours. After that, brain samples were transferred to Solution C for 60 hours at RT. Sections of the brain were prepared at 200 μm in thickness using Leica Cryostat CM1520 and collected on gelatin-coated slides (1% gelatin with 0.1% chromium potassium sulfate). Sections were left to dry at RT in the dark for up to 3 days. Dried sections were rinsed in MilliQ water twice. Sections were then incubated in 1:1:2 v/v Solution D:Solution E:MilliQ water for 10 minutes and rinsed with MilliQ water twice. Sections were then dehydrated by immersing in 50% ethanol, 75% ethanol, 95% ethanol, and absolute ethanol, sequentially. Finally, sections were cleared in xylene and mounted in DPX. Sections were left to dry in the dark at RT. Images were obtained using LSM710 Confocal Microscope (Carl Zeiss, Oberkochen, Germany). All images were realigned using StackReg plugin from Fiji. Neurite tracing was done with NeuronStudio [76] and then processed in ImageJ using Simple Neurite Tracing Plugin. Scholl analysis was performed using Simple Neurite Tracing Plugin from Fiji.

### Statistical analysis

Statistical differences of brain weights (Fig 1A and 1C), Sholl analysis (Fig 1F), total neurite lengths (Fig 1G), cortical layers and thickness (S3C–S3G and S4D–S4H Figs), Srebp1 isoforms (S8 Fig), and uptake of LPC-[^14^C]DHA between genotypes (S9F Fig), luciferase assays (Fig 5D and 5E), and in vitro lipogenesis assay (Fig 5G) were calculated using an unpaired *t* test. Statistical difference of brain weights between genotypes (Fig 2A and 2C, and S9C Fig), lipidomic analysis (Fig 2B and 2D, S5, S6 and S9D Figs), and normalized counts from nCounter gene expression assay (S7 Fig) were calculated using a Mann–Whitney test. Statistical differences between NSC^WT^ and NSC^KO^ with LPC-DHA treatment (Fig 6A–6C and S11 Fig) were calculated using a 2-way ANOVA followed by a Tukey multiple comparison test. All graphs and statistical tests were carried out on GraphPad Prism 7.0 and R 3.4.0. A *p*-value <0.05 was considered to be significant.

## Supporting information

S1 Table Key resources table. This table provides a list of reagents and resources used in this manuscript.
(XLSX)

S1 Data Primary dataset. This file contains numerical data that were used to generate figures in this manuscript.
(XLSX)

S1 Fig Cell type–specific expression of Mfsd2a in mouse brain. (A) In addition to endothelial cells, Mfsd2a is expressed to lower levels in newly formed oligodendrocytes, oligodendrocyte precursor cells, and astrocytes. RNA-seq data represented as FPKM are derived from Zhang and colleagues [28] and found at https://web.stanford.edu/group/barres_lab/brain_rnaseq.html. (B) Single cell sequencing analysis across the entire brain vascular system indicates that Mfsd2a is highly expressed in BBB capillary endothelium and to lower levels in oligodendrocytes and astrocytes. These data are obtained from http://betsholtzlab.org/VascularSingleCells/database.html and were originally reported by Vanlandewijck and colleagues [29]. a, arterial; aa, arteriolar; AC, astrocyte; capil, capillary; BBB, blood-brain barrier; EC, endothelial cell; FB, vascular fibroblast-like cell; FPKM, fragments per kilobase million; Mfsd2a, Major Facilitator Superfamily Domain containing 2a; MG, microglia; OL, oligodendrocyte; PC, pericytes; SMC, smooth muscle cell; v, venous; 1,2,3, subtypes.
(TIF)

S2 Fig Deletion of Mfsd2a in BBB endothelium of 2aiECKO mice. (A) Treatment scheme to obtain tamoxifen-induced postnatal deletion of Mfsd2a in BBB endothelium in 2aiECKO mice. P0 pups were injected with 50 μg/g body weight 4-OHT for 3 consecutive days. Brains were harvested at 4 weeks of age. (B) Immunofluorescence imaging indicated reduced Mfsd2a expression in cortical brain vasculature of tamoxifen-treated 4-week-old 2aiECKO relative to 2a^*fl/fl*^ mice. Cortical sections were stained with Hoechst, Glut1 (a BBB endothelial marker) and Mfsd2a. Scale bar, 100 μm.
(TIF)

S3 Fig Cortical layer analysis in brains of P7 2aKO mice. Brain coronal sections of P7 2aKO relative to age-matched WT stained with Hoechst and (A) Cux1 (Cortical layer II–IV marker) and (B) Ctip2 (Cortical layer V marker). Scale bar, 200 μm. Quantification of (C) layer I, (D) layer II–IV, (E) layer V, and (F) layer VI of P7 WT and 2aKO as a percentage of total cortical thickness. A small but significant increase is seen in layers II–IV and reduced thickness is seen in layer VI. Data are represented as mean ± SE. WT, *n* = 4; 2aKO, *n* = 4. *p < 0.05. (G) Quantification of cortical thickness indicate a significantly smaller cortical layer in P7 2aKO relative to age-matched WT. Data are represented as mean ± SE. WT, *n* = 4; 2aKO, *n* = 4. *p < 0.05. Numerical values underlying panels S3C–G can be found in S1 Data.
(TIF)

S4 Fig Cortical layer analysis in brains of e18.5 2aKO mice. Brain coronal sections of e18.5 2aKO relative to age-matched WT mice stained with Hoechst and (A) Cux1 (Cortical layer II–IV marker), (B) Ctip2 (Cortical layer V marker), and (C) Tbr1 (Cortical layer VI marker). Scale bar, 200 μm. Quantification of (D) layer I, (E) layers II–IV, (F) layer V, and (G) layer VI of indicated no change in cortical thickness in e18.5 2aKO compared to age-matched WT. Data are represented as mean ± SE. WT, *n* = 3; 2aKO, *n* = 4. (H) Quantification of cortical thickness indicated no significant difference between e18.5 2aKO relative to age-matched WT. Data are represented as mean ± SE. WT, *n* = 3; 2aKO, *n* = 4. Numerical values underlying panels S4D–H can be found in S1 Data.
(TIF)

S5 Fig Targeted lipidomic analysis of P8 2a^*fl/fl*^ and 2aECKO mice. Percentage of saturated, mono-, or polyunsaturated fatty acid species in PC (A), PE (B), and (PS) (C) phospholipid species in brain, represented as heatmaps. Fatty acid identity is designated as number of carbons:number of double bonds; e.g., 38:6 indicates a phospholipid with 38 carbons and 6 double bonds. Capital letters above each lane represent biological replicates of indicated genotype (*n* = 5 for 2a^*fl/fl*^ and 2aECKO). Scale bar represents percent PC, PE, or PS over total brain phospholipids. **p < 0.01; *p < 0.05.
(TIF)

S6 Fig Targeted lipidomic analysis of P8 2a^*fl/fl*^ and 2aiECKO mice. Percentage of saturated, mono-, or polyunsaturated fatty acid species in PC (A), PE (B), and PS (C) phospholipid species in brain, represented as heatmaps. Fatty acid identity is designated as number of carbons:number of double bonds; e.g., 38:6 indicates a phospholipid with 38 carbons and 6 double bonds. Capital letters above each lane represent biological replicates of indicated genotype (2a^*fl/fl*^, *n* = 4; 2aiECKO, *n* = 5). Scale bar represents percent PC, PE, or PS over total brain phospholipids. *p < 0.05.
(TIF)

S7 Fig Confirmation of up-regulation of pathways using Nanostring analysis on brains of P8 or e18.5 mice. Representative gene targets from Srebp-1 and/or Srebp-2 pathway are shown. (A) Direct mRNA quantification by Nanostring analysis of Srebp-1 and Srebp-2 gene targets on brains from P8 2a^*fl/fl*^ and 2aECKO mice. Normalized counts are represented as mean ± SE. 2a^*fl/fl*^, *n* = 5; 2aECKO, *n* = 5; biological replicates. **p < 0.01; *p < 0.05. (B) Nanostring analysis of Srebp-1 gene targets on brains from P8 2a^*fl/fl*^ and 2aiECKO mice, in which deletion of Mfsd2a in the BBB was induced by daily injections of 4-OHT injections from P0 to P3. Normalized counts are represented as mean ± SE; 2a^*fl/fl*^, *n* = 5; 2aiECKO, n = 5; biological replicates. **p < 0.01; *p < 0.05. (C) Nanostring analysis of Srebp-1 and Srebp-2 gene targets from brains from e18.5 2a^*fl/fl*^ and 2aECKO mice. Normalized counts are represented as mean ± SE; 2a^*fl/fl*^, *n* = 5; 2aECKO, *n* = 5; biological replicates. **p < 0.01. Experimental data depicted in this figure can be found in S1 Data.
(TIF)

S8 Fig Srebp-1c is the predominant Srebp-1 isoform induced in 2aECKO brains. Quantification of Srebp-1a and Srebp-1c isoforms by qRT-PCR in brains from P8 2a^*fl/fl*^ and 2aECKO mice indicate that Srebp-1c is the predominant isoform of Srebp expressed in brains of 2a^*fl/fl*^ mice and the predominant isoform that is up-regulated in brains of 2aECKO mice. *n* = 2, biological replicates. Three technical replicates were carried out for each sample. Experimental data depicted in this figure can be found in S1 Data.
(TIFF)

S9 Fig Neural stem cells as an in vitro model for Mfsd2a and LPC-DHA function. Mfsd2a is expressed in the endothelium of the BBB and in cells surrounding the ventricle (labeled “v”), a major neurogenic niche in the brain. Coronal sections of e18.5 2aKO and WT brains stained with Hoechst, (A) Glut1 (a BBB endothelial marker) and Mfsd2a, and (B) Nes (neural stem/progenitor marker) and Mfsd2a. Mfsd2a expression in cells near the ventricle is indicated by white arrows. Scale bar (A) 50 μm, (B) 100 μm. (C) An NSC deficiency model of Mfsd2a was generated using a floxed allele of Mfsd2a (2a^*fl/fl*^) crossed to NSC-cre driver Nes (2aNKO). Brain weights of P8 2aNKO are similar to 2a^*fl/fl*^ mice. Data are represented as mean ± SE; 2a^*fl/fl*^, *n* = 4; 2aNKO, *n* = 4. (D) Targeted lipidomic analysis of brains from P8 2a^*fl/fl*^ and 2aNKO mice. Percentage PC, PE, and PS containing DHA shown and represented as mean ± SE; 2a^*fl/fl*^, *n* = 4; 2aNKO, *n* = 5; biological replicates. *p < 0.05. (E) Western blot analysis of Mfsd2a expression in NSC^WT^ and NSC^KO^ whole cell lysate indicated that Mfsd2a is expressed in NSC^WT^ but not NSC^KO^ cells. β-actin served as a loading control. NSC^WT^, *n* = 2; NSC^KO^, *n* = 2; biological replicates. (F) LPC-[^14^C]DHA transport assay demonstrated a significant reduction in uptake of radiolabeled LPC-[^14^C]DHA relative to NSC^WT^ cells, indicating Mfsd2a function in NSC^WT^ cells. NSCs were treated with LPC-[^14^C]DHA for 30 minutes before DPM was quantified by scintillation counting. Uptake is expressed as mean ± SE. Technical replicates were carried out for each genotype. Numerical values underlying panels S9C, D, and F can be found in S1 Data.
(TIF)

S10 Fig Confirmation of Mfsd2a expression in NSC^KO^ complemented with Mfsd2a WT or Mfsd2a D96A adenovirus and Western blot analysis of Srebp-2. (A) Western blot analysis confirmed Mfsd2a expression in NSC^KO^ cells complemented with Mfsd2a WT or Mfsd2a D96A adenovirus with or without LPC-DHA treatment. β-actin served as a loading control. Non-virus-transduced NSC^KO^ controls, *n* = 3; NSC^KO^ Mfsd2a WT, *n* = 3; NSC^KO^ Mfsd2a D96A, *n* = 3; biological replicates. (B) Western blot analysis of Srebp-2 expression in NSC^WT^ cells treated with or without the indicated activators or inhibitors of Srebp processing or expression. Cell lysates were fractionated into nuclear and cytoplasmic/membrane fractions. Mev and 25-HC treatment resulted in the expected increased and decreased nSrebp-2 levels, respectively. LPC-DHA treatment did not reduce nSrebp-2 levels. β-actin served as a loading control. Numerical values underlying panel S10B can be found in S1 Data. GSK, GSK2033; Mev, Mevastatin plus mevalonic acid; T0, T0901317; 25-HC, 25-hydroxycholesterol plus cholesterol.
(TIF)

S11 Fig Targeted lipidomic analysis of NSC^WT^ and NSC^KO^ cells treated with or without LPC-DHA. Percentage of saturated, mono-, or polyunsaturated fatty acid species in PC (A), PE (B), and PS (C) phospholipid species in NSC^WT^ or NSC^KO^ with or without LPC-DHA treatment, represented as heatmaps. Fatty acid identity is designated as number of carbons:number of double bonds; e.g., 38:6 indicates a phospholipid with 38 carbons and 6 double bonds. Capital letters above each lane represent biological replicates of NSC^WT^ or NSC^KO^ with or without LPC-DHA treatment (*n* = 3 for NSC^WT^ control, NSC^WT^ plus LPC-DHA, NSC^KO^ control and NSC^KO^ plus LPC-DHA). Scale bar represents percent PC, PE, or PS over total NSC phospholipids. ****/####*p* < 0.0001; ***/###*p* = 0.0002; **/##*p* = 0.0021; */#*p* = 0.0332 (“*” for NSC^WT^; “#” for NSC^KO^). Ctrl, Control; PC, phosphatidylcholine; PE, phosphatidylethanolamine; PS, phosphatidylserine.
(TIF)

## Abbreviations

2aECKO: endothelial specific Mfsd2a knockout mouse
2a^*fl/fl*^: floxed allele of Mfsd2a
2aiECKO: tamoxifen-inducible endothelial specific Mfsd2a knockout mouse
2aKO: Mfsd2a knockout mouse
AA: arachidonic acid
BBB: blood-brain barrier
BRB: blood-retinal barrier
Cav-1: Caveolin-1
ChIPseq: chromatin immunoprecipitation sequencing
DHA: docosahexaenoic acid
e: embryonic day
ER: endoplasmic reticulum
HMG-CoA: 3-hydroxy-3-methylglutaryl coenzyme A
IHC: immunohistochemistry
Insig-1: insulin-induced gene 1
LC-MS/MS: liquid chromatography-tandem mass spectrometry
LPC: lysophosphatidylcholine
LPCAT3: lysophosphatidylcholine acyltransferase 3
LPC-DHA: DHA esterified to lysophosphatidylcholine
LXR: Liver X Receptor
Mfsd2a: Major Facilitator Superfamily Domain containing 2a
MOI: multiplicity of infection
MRM: multiple reaction monitoring
MS: mass spectrometric
NSC: neural stem/progenitor cell
P: postnatal day
PBS: phosphate buffered saline
PC: phosphatidylcholine
PE: phosphatidylethanolamine
PFA: paraformaldehyde
PS: phosphatidylserine
pSynSRE: 3-hydroxy-3-methylglutaryl coenzyme A synthase promoter
PUFA: polyunsaturated fatty acid
qRT-PCR: quantitative reverse transcriptase-PCR
RMA: robust multichip averaging
RT: room temperature
Scap: sterol cleavage-activating protein
SRE: sterol regulatory element
SREBP: sterol regulatory-element binding protein
TLC: thin-layer chromatography
WT: wild-type
