# Supplementary figures and images for: The lysolipid transporter Mfsd2a regulates lipogenesis in the developing brain
Source: PLoS Biol. 2018 Aug 3;16(8):e2006443. doi: 10.1371/journal.pbio.2006443 (PMC6093704; doi:10.1371/journal.pbio.2006443)

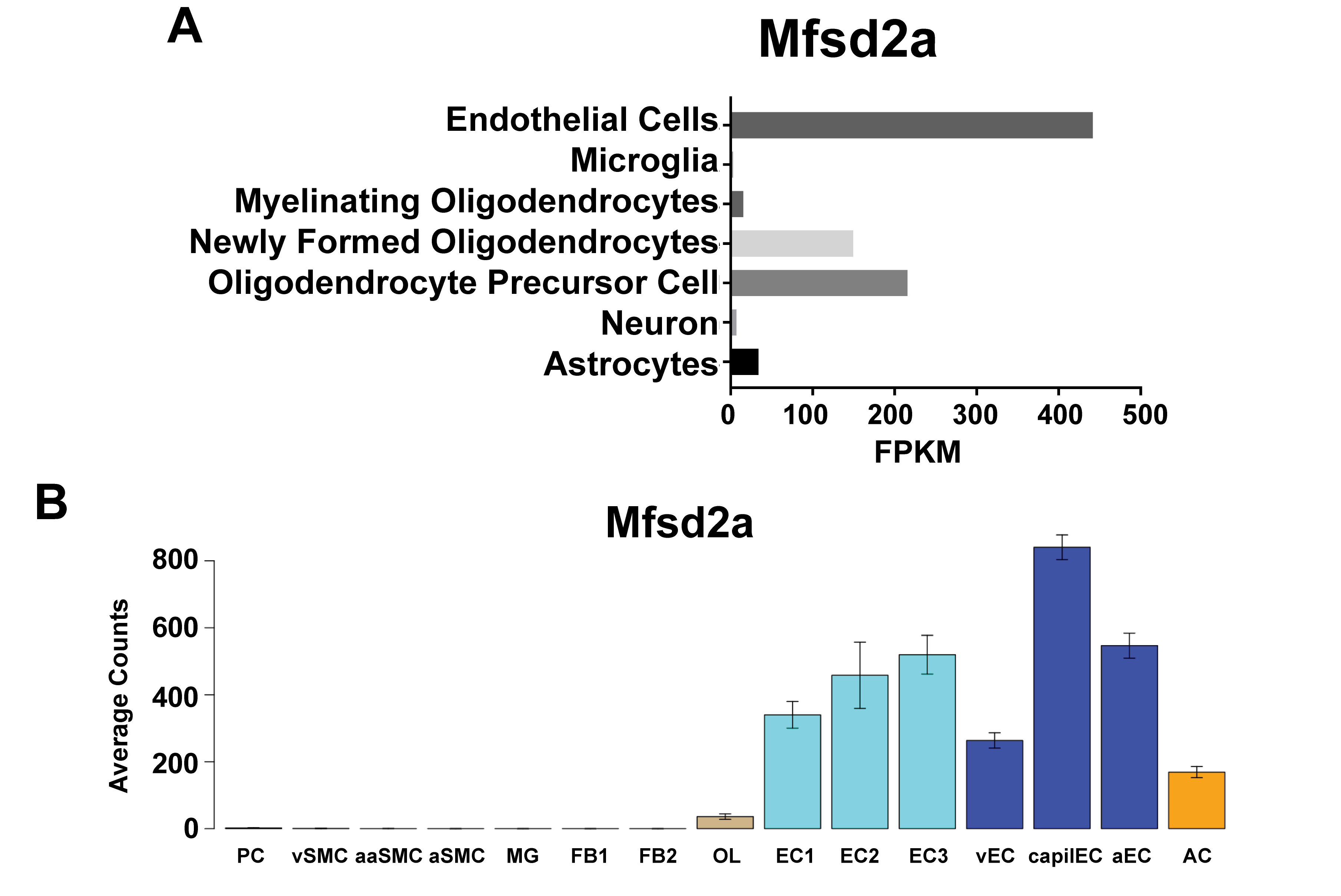

Supplement: S1 Fig — (A) In addition to endothelial cells, Mfsd2a is expressed to lower levels in newly formed oligodendrocytes, oligodendrocyte precursor cells, and astrocytes. RNA-seq data represented as FPKM are derived from Zhang and colleagues [28] and found at https://web.stanford.edu/group/barres_lab/brain_rnaseq.html. (B) Single cell sequencing analysis across the entire brain vascular system indicates that Mfsd2a is highly expressed in BBB capillary endothelium and to lower levels in oligodendrocytes and astrocytes. These data are obtained from http://betsholtzlab.org/VascularSingleCells/database.html and were originally reported by Vanlandewijck and colleagues [29]. a, arterial; aa, arteriolar; AC, astrocyte; capil, capillary; BBB, blood-brain barrier; EC, endothelial cell; FB, vascular fibroblast-like cell; FPKM, fragments per kilobase million; Mfsd2a, Major Facilitator Superfamily Domain containing 2a; MG, microglia; OL, oligodendrocyte; PC, pericytes; SMC, smooth muscle cell; v, venous; 1,2,3, subtypes. (TIF) [file pbio.2006443.s003.tif]

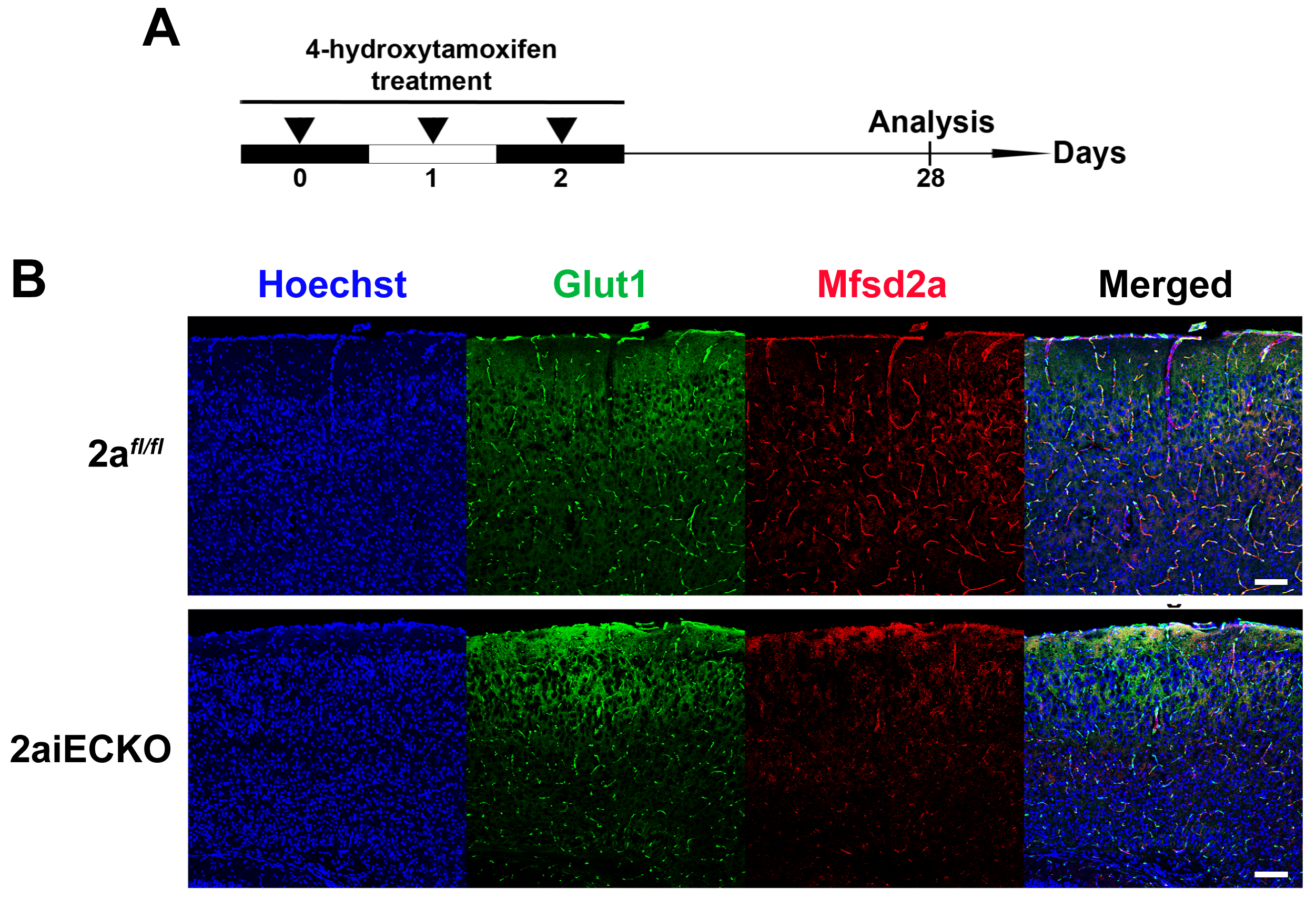

Supplement: S2 Fig — (A) Treatment scheme to obtain tamoxifen-induced postnatal deletion of Mfsd2a in BBB endothelium in 2aiECKO mice. P0 pups were injected with 50 μg/g body weight 4-OHT for 3 consecutive days. Brains were harvested at 4 weeks of age. (B) Immunofluorescence imaging indicated reduced Mfsd2a expression in cortical brain vasculature of tamoxifen-treated 4-week-old 2aiECKO relative to 2afl/fl mice. Cortical sections were stained with Hoechst, Glut1 (a BBB endothelial marker) and Mfsd2a. Scale bar, 100 μm. (TIF) [file pbio.2006443.s004.tif]

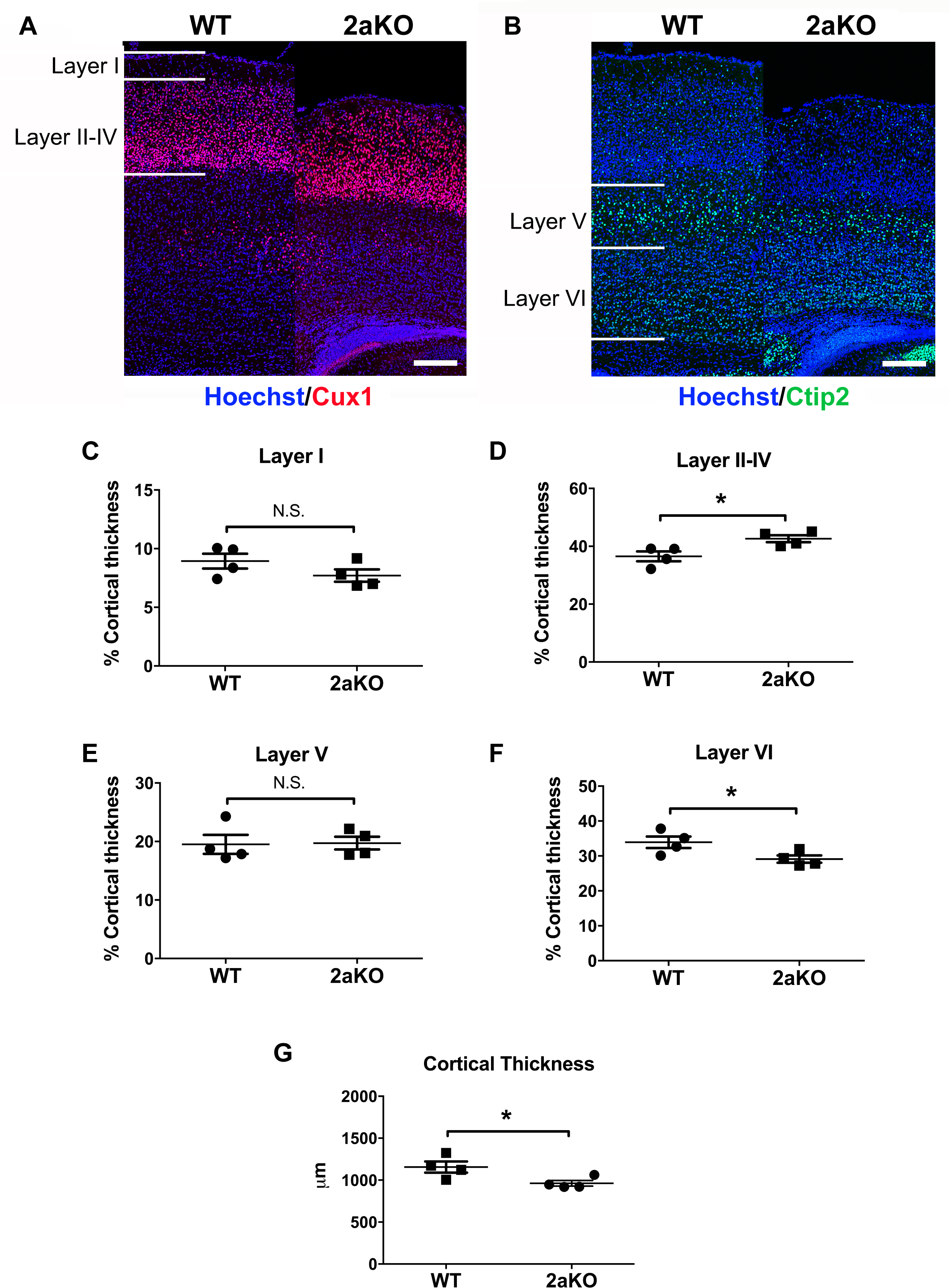

Supplement: S3 Fig — Brain coronal sections of P7 2aKO relative to age-matched WT stained with Hoechst and (A) Cux1 (Cortical layer II–IV marker) and (B) Ctip2 (Cortical layer V marker). Scale bar, 200 μm. Quantification of (C) layer I, (D) layer II–IV, (E) layer V, and (F) layer VI of P7 WT and 2aKO as a percentage of total cortical thickness. A small but significant increase is seen in layers II–IV and reduced thickness is seen in layer VI. Data are represented as mean ± SE. WT, n = 4; 2aKO, n = 4. *p < 0.05. (G) Quantification of cortical thickness indicate a significantly smaller cortical layer in P7 2aKO relative to age-matched WT. Data are represented as mean ± SE. WT, n = 4; 2aKO, n = 4. *p < 0.05. Numerical values underlying panels S3C–G can be found in S1 Data. (TIF) [file pbio.2006443.s005.tif]

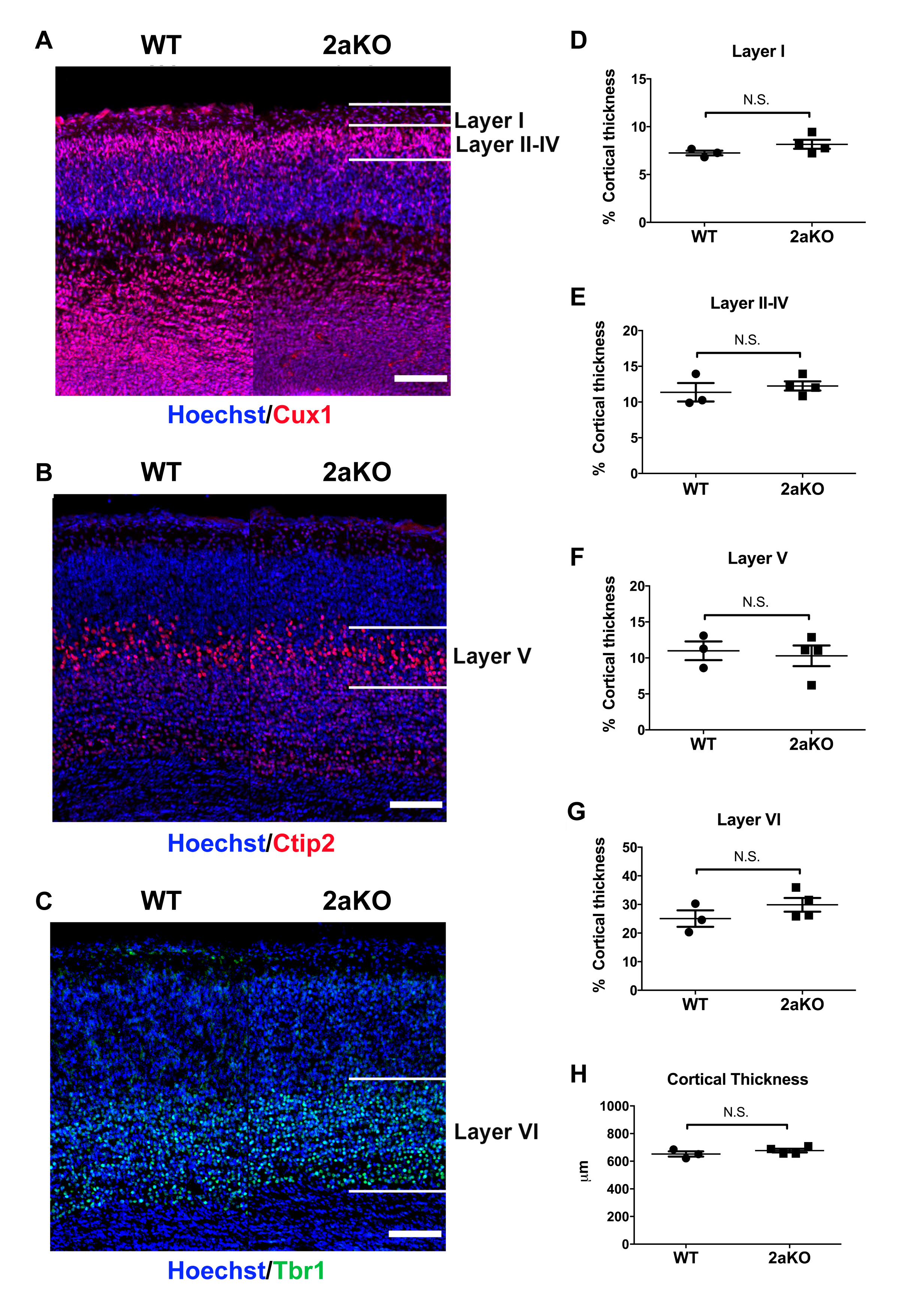

Supplement: S4 Fig — Brain coronal sections of e18.5 2aKO relative to age-matched WT mice stained with Hoechst and (A) Cux1 (Cortical layer II–IV marker), (B) Ctip2 (Cortical layer V marker), and (C) Tbr1 (Cortical layer VI marker). Scale bar, 200 μm. Quantification of (D) layer I, (E) layers II–IV, (F) layer V, and (G) layer VI of indicated no change in cortical thickness in e18.5 2aKO compared to age-matched WT. Data are represented as mean ± SE. WT, n = 3; 2aKO, n = 4. (H) Quantification of cortical thickness indicated no significant difference between e18.5 2aKO relative to age-matched WT. Data are represented as mean ± SE. WT, n = 3; 2aKO, n = 4. Numerical values underlying panels S4D–H can be found in S1 Data. (TIF) [file pbio.2006443.s006.tif]

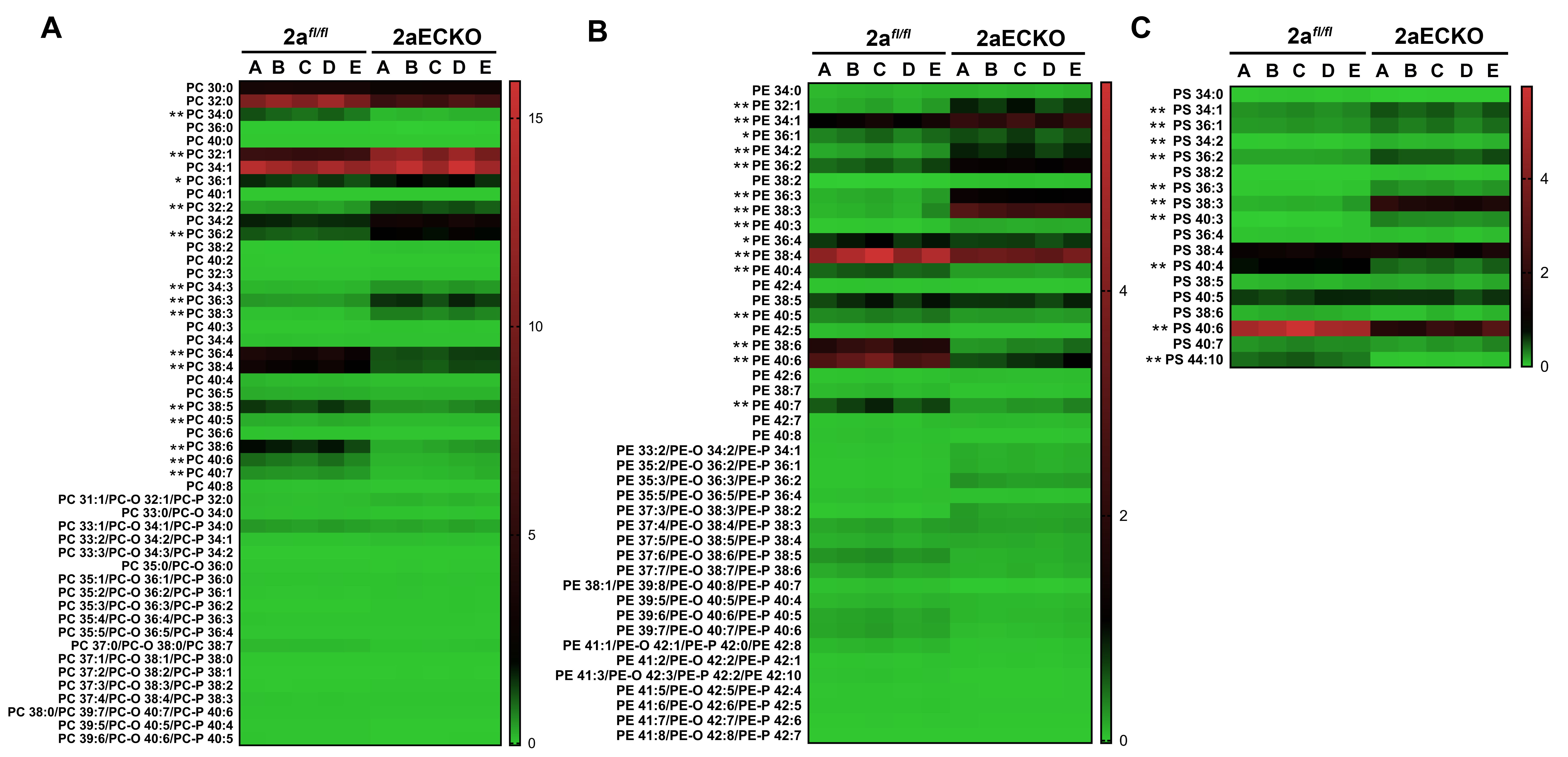

Supplement: S5 Fig — Percentage of saturated, mono-, or polyunsaturated fatty acid species in PC (A), PE (B), and (PS) (C) phospholipid species in brain, represented as heatmaps. Fatty acid identity is designated as number of carbons:number of double bonds; e.g., 38:6 indicates a phospholipid with 38 carbons and 6 double bonds. Capital letters above each lane represent biological replicates of indicated genotype (n = 5 for 2afl/fl and 2aECKO). Scale bar represents percent PC, PE, or PS over total brain phospholipids. **p < 0.01; *p < 0.05. (TIF) [file pbio.2006443.s007.tif]

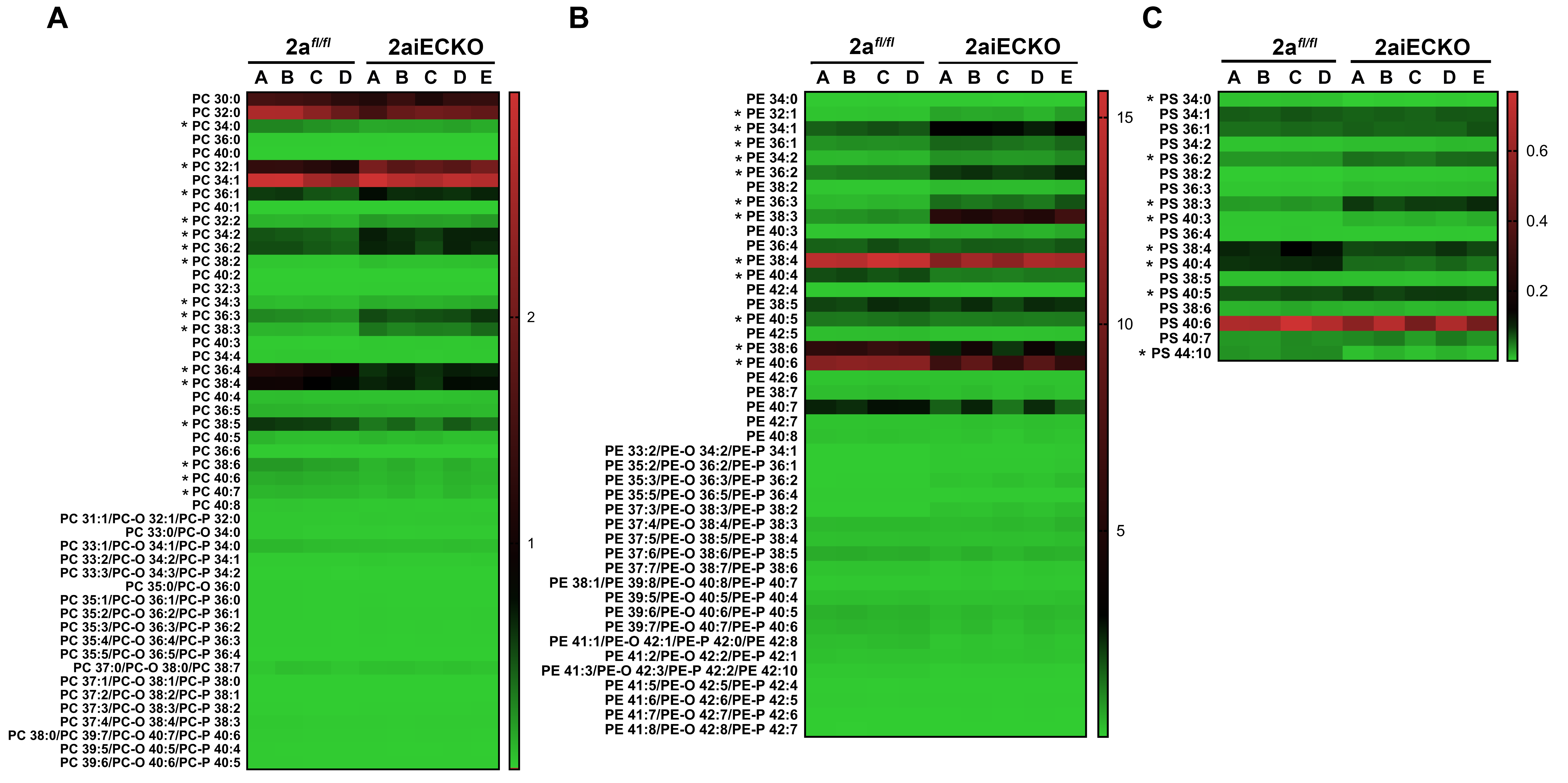

Supplement: S6 Fig — Percentage of saturated, mono-, or polyunsaturated fatty acid species in PC (A), PE (B), and PS (C) phospholipid species in brain, represented as heatmaps. Fatty acid identity is designated as number of carbons:number of double bonds; e.g., 38:6 indicates a phospholipid with 38 carbons and 6 double bonds. Capital letters above each lane represent biological replicates of indicated genotype (2afl/fl, n = 4; 2aiECKO, n = 5). Scale bar represents percent PC, PE, or PS over total brain phospholipids. *p < 0.05. (TIF) [file pbio.2006443.s008.tif]

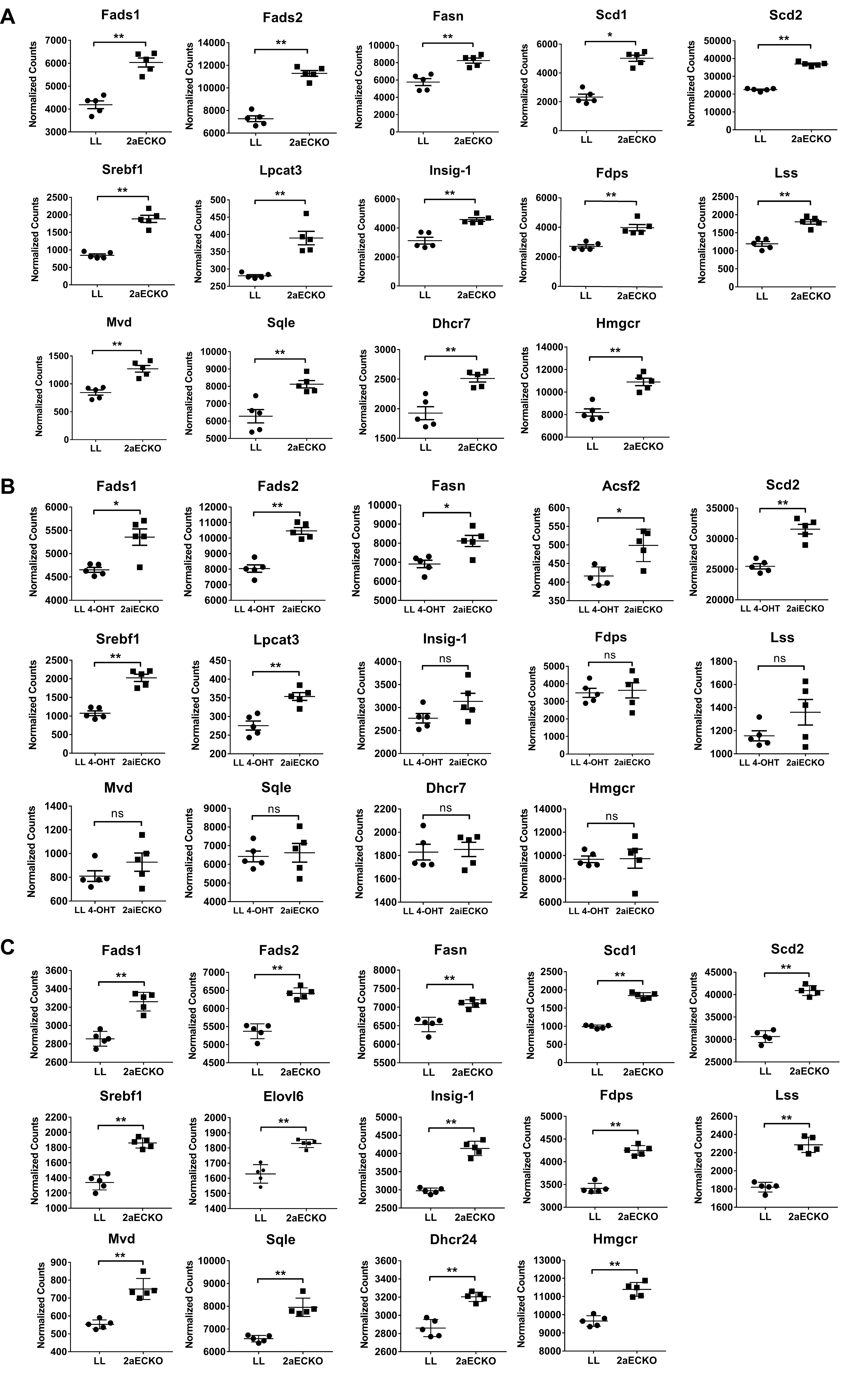

Supplement: S7 Fig — (A) Direct mRNA quantification by Nanostring analysis of Srebp-1 and Srebp-2 gene targets on brains from P8 2afl/fl and 2aECKO mice. Normalized counts are represented as mean ± SE. 2afl/fl, n = 5; 2aECKO, n = 5; biological replicates. **p < 0.01; *p < 0.05. (B) Nanostring analysis of Srebp-1 gene targets on brains from P8 2afl/fl and 2aiECKO mice, in which deletion of Mfsd2a in the BBB was induced by daily injections of 4-OHT injections from P0 to P3. Normalized counts are represented as mean ± SE; 2afl/fl, n = 5; 2aiECKO, n = 5; biological replicates. **p < 0.01; *p < 0.05. (C) Nanostring analysis of Srebp-1 and Srebp-2 gene targets from brains from e18.5 2afl/fl and 2aECKO mice. Normalized counts are represented as mean ± SE; 2afl/fl, n = 5; 2aECKO, n = 5; biological replicates. **p < 0.01. Experimental data depicted in this figure can be found in S1 Data. (TIF) [file pbio.2006443.s009.tif]

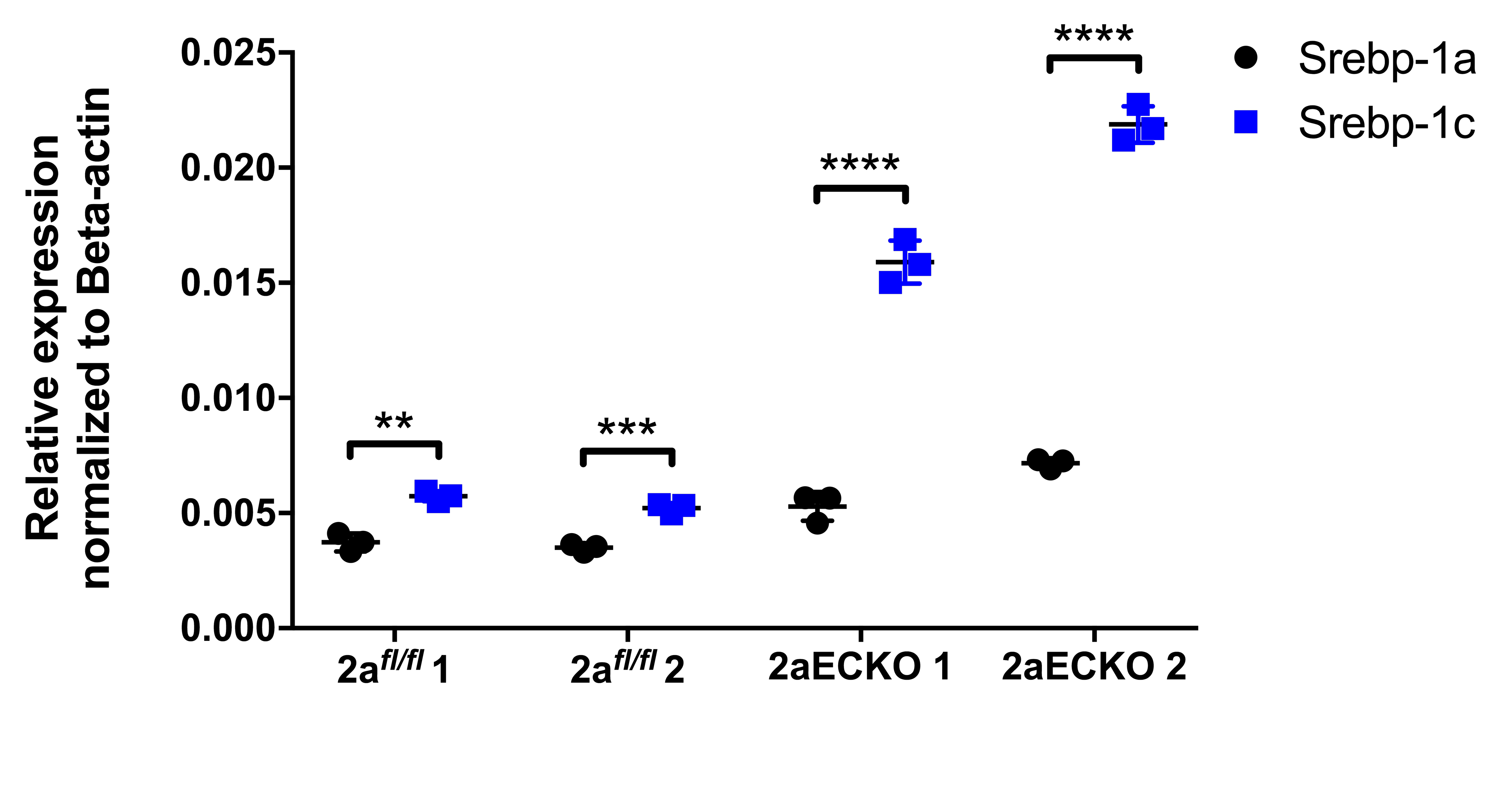

Supplement: S8 Fig — Quantification of Srebp-1a and Srebp-1c isoforms by qRT-PCR in brains from P8 2afl/fl and 2aECKO mice indicate that Srebp-1c is the predominant isoform of Srebp expressed in brains of 2afl/fl mice and the predominant isoform that is up-regulated in brains of 2aECKO mice. n = 2, biological replicates. Three technical replicates were carried out for each sample. Experimental data depicted in this figure can be found in S1 Data. (TIFF) [file pbio.2006443.s010.tiff]

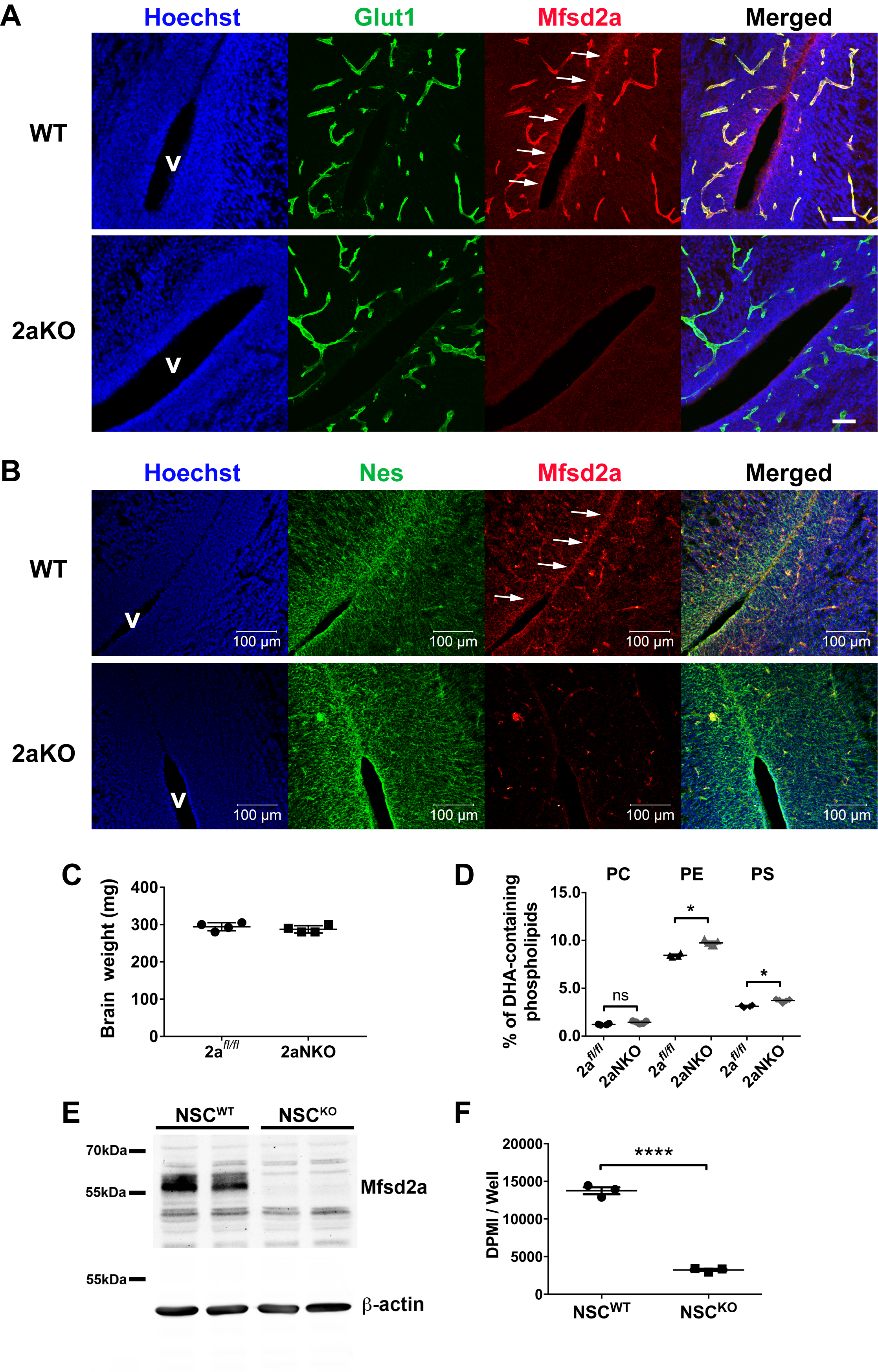

Supplement: S9 Fig — Mfsd2a is expressed in the endothelium of the BBB and in cells surrounding the ventricle (labeled “v”), a major neurogenic niche in the brain. Coronal sections of e18.5 2aKO and WT brains stained with Hoechst, (A) Glut1 (a BBB endothelial marker) and Mfsd2a, and (B) Nes (neural stem/progenitor marker) and Mfsd2a. Mfsd2a expression in cells near the ventricle is indicated by white arrows. Scale bar (A) 50 μm, (B) 100 μm. (C) An NSC deficiency model of Mfsd2a was generated using a floxed allele of Mfsd2a (2afl/fl) crossed to NSC-cre driver Nes (2aNKO). Brain weights of P8 2aNKO are similar to 2afl/fl mice. Data are represented as mean ± SE; 2afl/fl, n = 4; 2aNKO, n = 4. (D) Targeted lipidomic analysis of brains from P8 2afl/fl and 2aNKO mice. Percentage PC, PE, and PS containing DHA shown and represented as mean ± SE; 2afl/fl, n = 4; 2aNKO, n = 5; biological replicates. *p < 0.05. (E) Western blot analysis of Mfsd2a expression in NSCWT and NSCKO whole cell lysate indicated that Mfsd2a is expressed in NSCWT but not NSCKO cells. β-actin served as a loading control. NSCWT, n = 2; NSCKO, n = 2; biological replicates. (F) LPC-[14C]DHA transport assay demonstrated a significant reduction in uptake of radiolabeled LPC-[14C]DHA relative to NSCWT cells, indicating Mfsd2a function in NSCWT cells. NSCs were treated with LPC-[14C]DHA for 30 minutes before DPM was quantified by scintillation counting. Uptake is expressed as mean ± SE. Technical replicates were carried out for each genotype. Numerical values underlying panels S9C, D, and F can be found in S1 Data. (TIF) [file pbio.2006443.s011.tif]

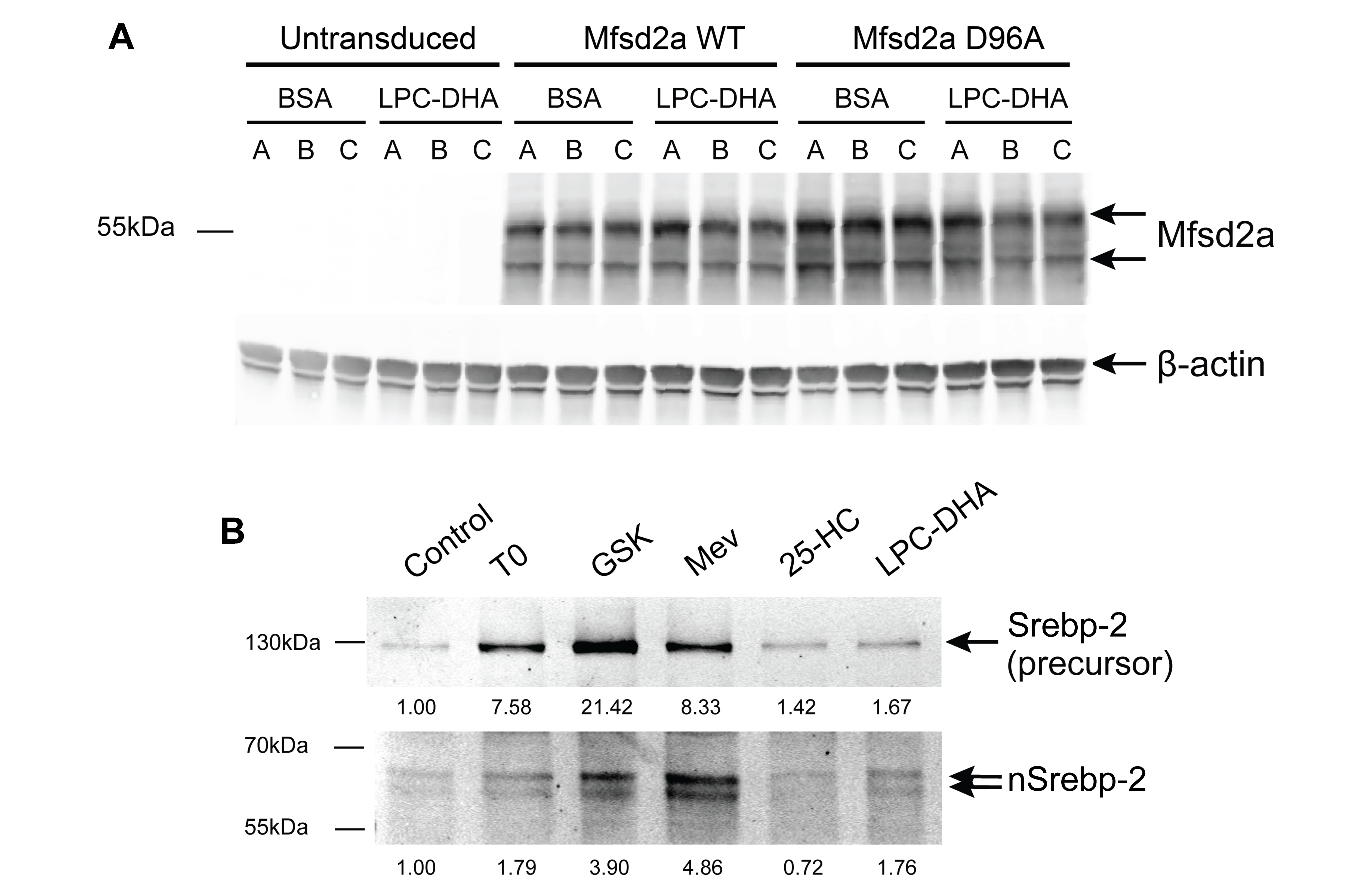

Supplement: S10 Fig — (A) Western blot analysis confirmed Mfsd2a expression in NSCKO cells complemented with Mfsd2a WT or Mfsd2a D96A adenovirus with or without LPC-DHA treatment. β-actin served as a loading control. Non-virus-transduced NSCKO controls, n = 3; NSCKO Mfsd2a WT, n = 3; NSCKO Mfsd2a D96A, n = 3; biological replicates. (B) Western blot analysis of Srebp-2 expression in NSCWT cells treated with or without the indicated activators or inhibitors of Srebp processing or expression. Cell lysates were fractionated into nuclear and cytoplasmic/membrane fractions. Mev and 25-HC treatment resulted in the expected increased and decreased nSrebp-2 levels, respectively. LPC-DHA treatment did not reduce nSrebp-2 levels. β-actin served as a loading control. Numerical values underlying panel S10B can be found in S1 Data. GSK, GSK2033; Mev, Mevastatin plus mevalonic acid; T0, T0901317; 25-HC, 25-hydroxycholesterol plus cholesterol. (TIF) [file pbio.2006443.s012.tif]

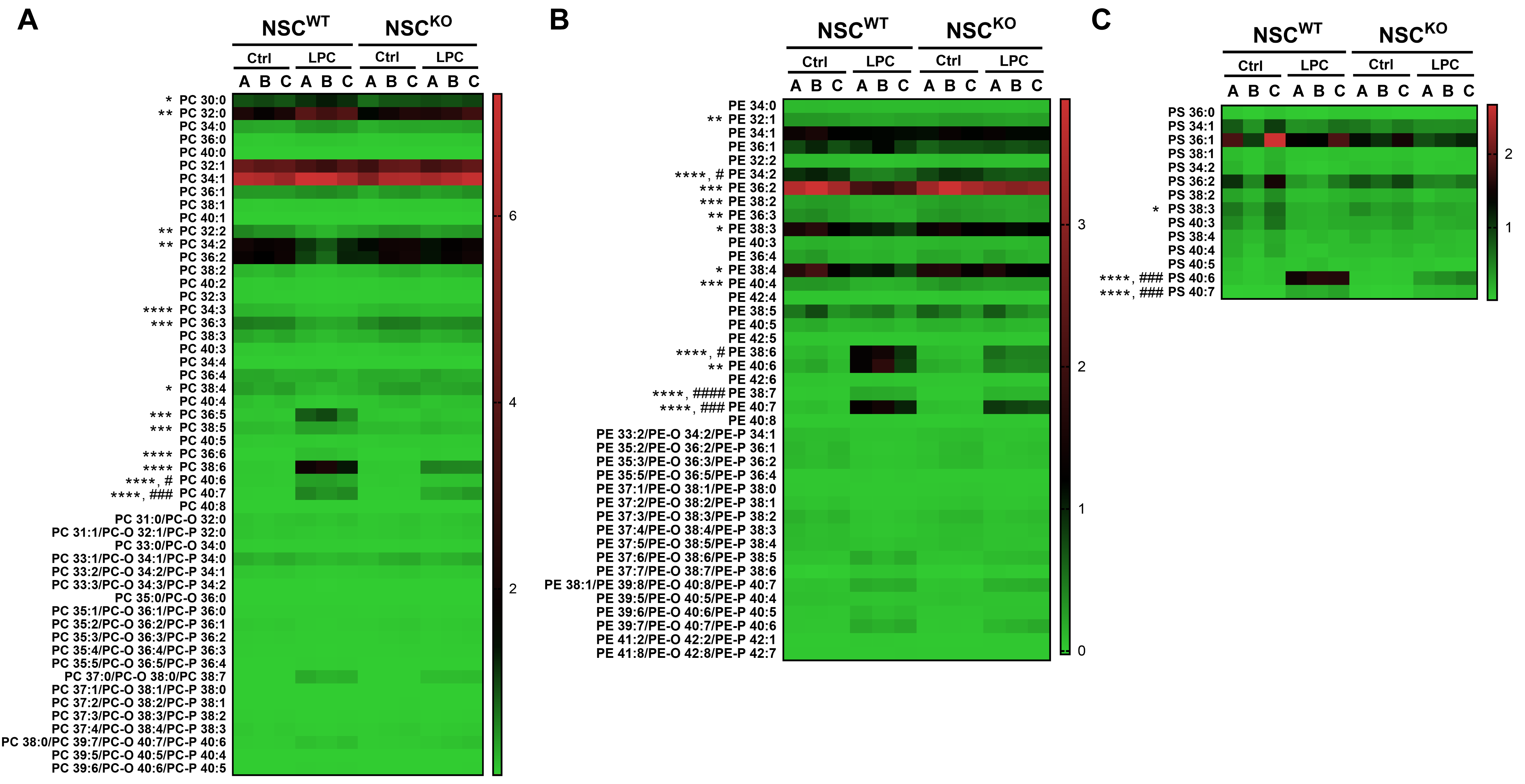

Supplement: S11 Fig — Percentage of saturated, mono-, or polyunsaturated fatty acid species in PC (A), PE (B), and PS (C) phospholipid species in NSCWT or NSCKO with or without LPC-DHA treatment, represented as heatmaps. Fatty acid identity is designated as number of carbons:number of double bonds; e.g., 38:6 indicates a phospholipid with 38 carbons and 6 double bonds. Capital letters above each lane represent biological replicates of NSCWT or NSCKO with or without LPC-DHA treatment (n = 3 for NSCWT control, NSCWT plus LPC-DHA, NSCKO control and NSCKO plus LPC-DHA). Scale bar represents percent PC, PE, or PS over total NSC phospholipids. ****/####p < 0.0001; ***/###p = 0.0002; **/##p = 0.0021; */#p = 0.0332 (“*” for NSCWT; “#” for NSCKO). Ctrl, Control; PC, phosphatidylcholine; PE, phosphatidylethanolamine; PS, phosphatidylserine. (TIF) [file pbio.2006443.s013.tif]
